# Transdermal entry of a non-pathogenic filamentous fungus *Aspergillus oryzae* induces an immunomodulatory response in skin-draining lymph nodes

**DOI:** 10.3389/fimmu.2026.1815568

**Published:** 2026-06-15

**Authors:** Thanh Dat Ta, Madoka Ozawa, Yuka Kasuga, Midori Shida, Yui Kotani, Haruko Hayasaka, Michio Tomura, Eiji Umemoto, Tomoya Katakai

**Affiliations:** 1 Department of Immunology, Niigata University Graduate School of Medical and Dental Sciences, Niigata, Japan; 2 School of Health Science, Faculty of Medicine, Niigata University, Niigata, Japan; 3 Laboratory of Immune Molecular Function, Faculty of Science and Engineering, Kindai University, Higashiosaka, Japan; 4 Laboratory of Immunology, Faculty of Pharmacy, Osaka Ohtani University, Tondabayashi, Japan; 5 Laboratory of Microbiology and Immunology, University of Shizuoka, Shizuoka, Japan

**Keywords:** allergic responses, *Aspergillus oryzae*, B cells, beta-glucan, skin-draining lymph node, type 2 resident dendritic cells

## Abstract

**Introduction:**

The immune response to non-pathogenic fungi entering the skin remains largely unknown. In this study, we focused on the fermentative filamentous fungus *Aspergillus oryzae* (Ao), which is traditionally utilized in Japan.

**Methods:**

Mice were subcutaneously inoculated with the Ao conidia and skin-draining lymph nodes were harvested over time for flow cytometry to evaluate various immune cell subsets. Quantitative reverse transcription-PCR was also conducted to assess the cytokine expression (IFN-γ, IL-4, IL-6, IL-10, IL-12, IL-17, TGF-β, TNF-α) at 4 or 7 days after Ao inoculation. To detect antigen-specific antibody production, mice were immunized with Ao and/or ovalbumin in aluminum hydroxide adjuvant, and sera were collected at day -1, 14, and 35 for ELISA. Fluorescence immunohistostaining was employed to visualize the structural reorganization of the lymph nodes. Active cutaneous anaphylaxis reaction was assessed for the impact of Ao inoculation on allergic response.

**Results:**

We observed a marked enlargement of skin-draining lymph nodes and increased immune cell numbers within days. Notably, this was characterized by a marked increase in the number of activated B cells and type 2 resident dendritic cells. These responses were partially reproduced by Ao cell wall components and purified β-glucans; the Ao-dependent downregulation of Dectin-1 expression on dendritic cells supports these findings. However, live Ao elicited the most pronounced lymph node response, as heat inactivation and the cell wall fraction clearly attenuated it, suggesting that other components are also necessary. Ao appears to induce a relatively mild response in the lymph nodes, characterized by a marked increase in IL-4 expression, whereas other cytokines were suppressed or unaltered. In the long term, Ao entry elicited immune memory with antibody production specific to the conidial proteins and β-glucan, although it did not enhance the antibody against another antigen/adjuvant. Furthermore, we found that pre-inoculation with Ao inhibited allergic responses. This is consistent with the novel effects of Ao inoculation, which reduces the CD301b^+^ dendritic cells in lymph nodes while increases their PD-L1 expression.

**Conclusion:**

Transdermal Ao entry clearly induces adaptive immunity in draining lymph nodes. However, the response is not necessarily intense and shows an immunomodulatory aspect with less harmful or even beneficial effects, potentially suppressing allergic diseases.

## 1 Introduction

Continuous contact with diverse environmental and symbiotic microorganisms is thought to stimulate and strengthen the immune system, while controlling it to function in a proper balance (1). The excessively hygienic living environment in modern society, built in less than the past century to prevent infectious diseases, has drastically reduced the quantity and diversity of microorganisms around us, distancing even those with beneficial effects on the body (2). Throughout evolutionary history, humans have lived in natural environments filled with numerous microorganisms, and our immune systems have been developed to adapt to these conditions. However, the reduction in microbial exposure due to rapid changes in living environments and lifestyles that accompany the development of modern society is likely to have caused immune dysfunction through its weakening and disturbance (3–5). The increasing prevalence of various immune-related disorders, such as allergies and autoimmune diseases, can be considered a reckless state of the immune system due to its dysregulation. Addressing this issue is extremely difficult; however, the surrounding microbial environment must be reconsidered. From this perspective, utilizing beneficial microorganisms traditionally used to produce various fermented foods has great potential.

A wide variety of fermented foods has been produced in Japan since ancient times (6–8). Microorganisms involved in the fermentation process are also abundant in such living environments (9–12), and it is assumed that frequent contact with them leads to their regular entry into the body. Not only are they ingested as food, but they also frequently enter the body via respiratory inhalation or small skin wounds, which potentially affect the immune system. *Aspergillus oryzae* (Ao, yellow koji mold), a fermenting microorganism essential for the production of traditional Japanese food ingredients and seasonings, such as miso, soy sauce, sake, rice vinegar, and pickles, has long been cultivated and utilized as a fermentation-capable filamentous fungus unique to Japan (6, 13, 14). *Aspergillus flavus*, a close relative and probable natural ancestor of Ao, is known as one of the pathogens causing aspergillosis and produces aflatoxin, an extremely potent mycotoxin (15). However, it has been demonstrated that Ao lacks the series of genes necessary for producing aflatoxin and is essentially a harmless non-pathogenic fungus (16–18). Once Ao enters the body, it is inevitably recognized as a foreign substance and is targeted for elimination by the host immune system (19). However, the nature of the immune response against this beneficial fungus remains largely unknown. Furthermore, our understanding of how contact with harmless microorganisms affects vaccination and immune disorders remains limited.

The adaptive immune response to microbes entering the body surface is primarily induced in regional draining lymph nodes (LNs); the mechanisms of typical responses to specific antigens have been elucidated to a certain extent (20, 21). However, the responses to individual microorganisms vary widely, and many aspects remain unclear. Moreover, research on non-pathogenic microorganisms is essentially insufficient, resulting in remarkably limited knowledge. There have been a few reports on the immune response to Ao (22, 23), although details regarding the LN response remain elusive.

In this study, we aimed to elucidate the immunological impact of non-pathogenic fermentative fungi *in vivo* by analyzing the detailed responses occurring in the superficial skin-draining LNs of mice subcutaneously inoculated with Ao. Transdermal entry of Ao induced clear responses in LNs within a few days, including tissue enlargement and various changes in immune cell subsets, in particular B cells. Nevertheless, these alterations were suggested to be not intense, but rather a type of regulatory response that is less harmful to the body and suppresses an allergic reaction. These findings indicate that Ao is a beneficial filamentous fungus that modulates the immune system to achieve an appropriate balance of responses via moderate stimuli, indicating promising prospects for its active utilization in the future.

## 2 Materials and methods

### 2.1 Mice

C57BL/6JJcl mice were purchased from CLEA Japan. Mice were maintained under specific pathogen-free conditions in the animal facility of Niigata University, and aged 8–12 weeks were used in the experiments. Most analyses of Ao inoculation used male mice. Although a small number of female mice were used as well, no clear differences were observed compared to the results obtained with male mice. Analyses for antibody production in serum were performed using female mice. Active cutaneous anaphylaxis assay was performed using male mice. All animal procedures were approved by the Committee on Animal Research at Niigata University.

### 2.2 Fungus culture and conidia preparation

*Aspergillus oryzae* RIB40 (14) was provided by the National Research Institute of Brewing (Hiroshima, Japan). Ao was grown on 5% Czapek-Dox Agar solid media (Sigma-Aldrich) at 30°C for 10 to 14 days until conidiophores/conidia were sufficiently formed. The Ao cultures were observed and microphotographed using a stereomicroscope SZX7 (Olympus) equipped with a CMOS camera WRAYCAM-NEO1600 (WRAYMER). The conidia of Ao (AoC) were collected by scraping off the culture plates with PBS using a scraper and passing them through a nylon mesh filter to remove hyphae and large debris. The conidial suspension was centrifuged at 3,500 rpm for 15 min, followed by washing with PBS. The number and concentration of conidia were determined by counting using a hemocytometer. The conidia were resuspended in PBS to a final concentration of 1 × 10^7^/100 µL (1 × 10^8^/1 mL) and stored at −80 °C until use.

For dry weight measurement, AoC suspension was heat-dried at 50–60 °C and weighed. One milligram of dry weight conidia corresponded to approximately 3.17 × 10^7^ conidia (~31.5 pg/conidium). In this study, the dose of 5 × 10^5^ conidia (0.15 µg) was used in all experiments.

Heat inactivation of AoC: AoC in PBS were incubated at various temperatures in a block incubator (ASTEC). For complete heat inactivation lacking growth ability, AoC was treated at 70 °C for 10 min.

Preparation of whole glucan particle (WGP): AoC was treated with 1 M NaOH at 80 °C for 2 h and washed with DW thrice. AoC was next treated with 1 M acetic acid at 80 °C for 2 h and washed with DW more than three times until pH becoming neutral. AoC was then suspended in PBS to a final concentration of 1 × 10^7^ particles/100 µL. To confirm the form of the particles, AoC-WGP, live AoC, and zymosan were stained with 12.5 µg/mL propidium iodide (Sigma) and 625 µg/mL aniline blue (Wako). Stained specimens were mounted on glass slides with cover glasses (Matsunami Glass Ind.) and examined using an FV1200 confocal microscope (Olympus).

Extraction of the AoC protein fraction and SDS-PAGE: 1 × 10^8^ AoC was suspended in 1 mL of ice-cold 50 mM of Tris-HCl, 2 mM of EDTA, 2% SDS, 1 mM of PMSF, Protease Inhibitor Cocktail set IV (Wako), and sonicated using a handy sonicator UR-20P (Tomy Seiko). The sonication was repeated cycles at 28kHz for 15 s and rest for 15 s on ice until the AoC suspension became clear. The suspension was centrifuged at 15,000 rpm for 10 min, and the supernatant was collected as the protein extract. The solution was dialyzed with PBS at 4°C for overnight and determined the protein concentration was quantified by Lowry method using DC Protein Assay Kit (BioRad), and stored at −30°C. The protein extract was mixed with 6× loading buffer and denatured at 95°C for 5 min, subsequently loaded onto 10% SDS-polyacrylamide gels for electrophoresis. The gels were stained with Coomassie Brilliant Blue (Bio-Rad Laboratories).

### 2.3 β-glucan associated reagents

Zymosan A (*Saccharomyces cerevisiae*) (Fujifilm Wako) was dissolved in PBS to a concentration of 5 mg/mL and boiled for 1 h followed by chilling at room temperature for 1 h (24). The zymosan was washed thrice with ice-cold PBS after centrifugation at 300 × *g* at 4 °C for 10 min and resuspended in PBS at 5 mg/mL. Zymosan solution was used for injection after dilution to 1 mg/mL with PBS. Curdlan (β1,3-glucan from *Alcaligenes faecalis* var. myxogenes) (Fujifilm Wako) was dissolved in 0.25 M NaOH for making alkali stock solution and stored at 4°C. To prepare the working solution, the alkali stock solution was diluted 10-fold with PBS and adjusted to pH 7 with 1 M HCl. Laminarin (β-1,3/1,6-glucan from *Laminaria digitata*) (InvivoGen) was dissolved in PBS at a concentration of 1 mg/mL.

### 2.4 Subcutaneous inoculation and isolation of skin-draining LNs

AoC or other substances (left side of the mouse) and PBS (right side) were subcutaneously injected into the fore footpad, hind footpad, foreleg, shoulder, chest, flank, lower abdomen, and tail base (8 sites/side). The inoculation sites were selected based on known lymphatic drainage pathways by which subcutaneously injected substances are transported to the respective skin-draining LNs analyzed in this study. 5 × 10^5^/20 µL AoC, 5 × 10^5^ particles/20 µL AoC-WGP, 20 µg/20 µL zymosan, 20 µg/20 µL curdlan, or 20 µg/20 µL laminarin were inoculated into each site. Superficial skin-draining LNs (axial, brachial, inguinal, and popliteal nodes) were excised for further analysis.

To assess the survival of the conidia following *in vivo* inoculation, subcutaneous tissues surrounding the inoculation site and regional draining-LN were excised 1 h, 4 days, and 7 days after inoculation. The tissue was minced, homogenized in PBS, and plated onto Czapek-Dox Agar solid medium. The appearance of colonies was observed on day 4.

### 2.5 Whole LN examination and size quantification

The excised LNs were soaked in PBS and examined under a stereomicroscope SZX7 (Olympus) equipped with a WRAYCAM-NEO1600 (WRAYMER). Light-field images were captured using Captman software (WRAYMER) and processed using Adobe Photoshop (Adobe Systems). For the size quantification of LNs from the images, Fiji (an open-source image-processing package based on ImageJ) software was used for image processing. The images were binarized by manual thresholding, and the pixels for the bright LN region against the dark background were measured.

### 2.6 Antibodies

The fluorochrome- or biotin-conjugated, or unconjugated primary antibodies used were as follows: APC-anti-CD3e (145-2C11), PE-anti-B220 (RA3-6B2), PE-anti-CD4 (RM4-5), FITC-anti-CD8 (53-6.7), PE-anti-MHC class II (M5/114.15.2), eF660-anti-F4/80 (BM8), APC-anti-Ly6C (HK1.4), and PE-anti-IgD (11-26c), biotin-anti-GL7 (GL-7), PE-anti-CD11c (N418), biotin-anti-CD274 (MIH5) [eBioscience]; biotin-anti-CD69 (H1.2F3), PE-anti-CD138 (281-2) [BD Pharmingen]; AF488- or APC-anti-B220 (RA3-6B2), APC-anti-CD11c (N418), Alexa Fluor (AF)488-anti-CD11b (M1/17), PE-anti-CD169 (3D6.112), APC-anti-CD301b (URA-1), APC-anti-CD38 (90), biotin-anti-mouse IgG1 (RMG1-1), biotin-anti-mouse IgD (11-26c.2a), and rat IgG2b isotype control (RTK4530) [BioLegend]; FITC-anti-CD25 (PC61.5.3) [Invitrogen]; biotin-anti-Dectin-1 (2A11) [BioRad]; anti-desmin (rabbit polyclonal) [Abcam]; biotin-anti-LYVE-1 (BAF2125, goat polyclonal) [R&D Systems]; anti-laminin (rabbit polyclonal) [LSL]. For secondary reagents, PE-, APC-, AF488-, or AF633-conjugated streptavidin, anti-rabbit IgG, and anti-goat IgG were purchased from Molecular Probes. PE-anti-FoxP3 (FJK-16s) [Invitrogen]; PE-anti-IFN-γ (XMG1.2), PE-anti-IL-10 (JES5-16E3) [BioLegend]; PE-anti-IL-4 (11B11), PE-anti-IL-17A (TC11-18H10), and PE-anti-TNF-α (MP6-XT22) [BD Pharmingen] were used for intracellular detection.

### 2.7 Flow cytometry

Single-cell suspensions were prepared from four excised skin-draining LNs (axillary, brachial, inguinal, and popliteal nodes) on one side of the mouse. LNs were cut into small fragments and digested with 1 mg/mL collagenase D and 0.1 mg/mL DNase I (Roche Diagnostics) as described (25), followed by counting the total cell numbers using a hemocytometer. The cells were then stained with fluorescently labeled antibodies. For intracellular cytokine detection, 2 × 10^6^ LN cells were stimulated with PMA (50 ng/mL) and inomycin (500 ng/mL) in the presence of 2 µM BD GolgiStop (BD Bioscience) in 8% FCS-RPMI 1640 medium at 37°C for 5 h. The cells were washed with medium, stained for the surface markers (CD4 and CD8) at 4°C for 30 min, and fixed and permeabilized using 1× BD Perm/Wash™ buffer (BD Bioscience). Intracellular cytokines were then stained with antibodies against IFN-γ, IL-4, IL-10, IL-17, and TNF-α at 4 °C for 20 min. For intracellular Foxp3 detection, cells were first stained with anti-CD4 and anti-CD25 antibodies, followed by fixation/permeabilization, and stained with anti-Foxp3 antibody at 4 °C for overnight. Data were acquired using a FACSCalibur flow cytometer (BD Biosciences) and analyzed using CellQuest (BD Biosciences) and FlowJo software. Dead cells were excluded using forward scatter and PI staining data.

### 2.8 Quantitative RT-PCR

Total RNA was extracted from cells enzymatically isolated from skin-draining LNs (axillary, brachial, inguinal, and popliteal nodes) using TRIzol reagent (Invitrogen), and first-strand cDNA was synthesized using PrimeScript II reverse transcriptase and oligo dT primer (Takara). Quantitative PCR was performed using a TB Green Premix Ex Taq™ II (Tli RNaseH Plus) (Takara) and LightCycler (Roche). Primers used were as follows: GAPDH, 5’-GCCAAGGTCATCCATGACAACT-3’ and 5’-GAGGGGCCATCCACAGTCTT-3’. IFN-γ, 5’-TTCTTCAGCAACAGCAAGGC-3’ and 5’-TCAGCAGCGACTCCTTTTCC-3’; IL-4, 5’-AACGAGGTCACAGGAGAAGG-3’ and 5’-TCTGCAGCTCCATGAGAACA-3’; IL-6, 5’-GAGGATACCACTCCCAACAGACC-3’ and 5’-AAGTGCATCATCGTTGTTCATACA-3’; IL-10, 5’-GGT​TGC​CAA​GCC​TTA​TCG​GA-3’ and 5’-ACC​TGC​TCC​ACT​GCC​TTG​CT-3’; IL-12p35, 5’-GATGACATGGTGAAGACGGC-3’ and 5’-AGGCACAGGGTCATCATCAA-3’; IL-17A, 5’-TCTCCACCGCAATGAAGACC-3’ and 5’- CACACCCACCAGCATCTTCT-3’; TGF-β1, 5’-TGCGCTTGCAGAGATTAAAA-3’ and 5’-CTGCCGTACAACTCCAGTGA-3’; and TNF-α, 5’-ATGAGGACAGAAAGCATGA-3’ and 5’-AGTAGACAGAAGAGCGTGGT-3’. The mRNA levels of the test genes were normalized to those of GAPDH.

### 2.9 Serum antibody production

Heat-inactivated AoC (5 x 10^5^) and/or 2 mg/mL ovalbumin (OVA, albumin from chicken egg white, A-5378, Sigma) in PBS were mixed with aluminum hydroxide (alum) adjuvant (Inject Alum, Thermo Fisher Scientific) and subcutaneously injected into mouse hind footpad at a dose of 20 μL/site (equivalent to 100 μg OVA). Blood was collected from the facial vein using a Goldenrod animal lancet (5 mm, MEDIpoint) on days -1, 14, 35 and centrifuged at 15,000 rpm at 4 °C for 10 min to obtain serum.

### 2.10 ELISA

Ninety-six well plates (9018, CORNING) were coated with 10 µg/mL AoC protein extract, 10 µg/mL curdlan, or 10 μg/mL OVA in PBS at 4 °C overnight and blocked with 1% bovine serum albumin in 0.05% Tween 20-PBS (PBST). Mouse serum was serially diluted (1:4) in PBS (4- or 6-steps) and added to the plate, then incubated at room temperature for 2 h. After washing thrice with PBST, the plate was incubated with a 1:10,000 or 1:20,000 dilution of HRP-conjugated Goat anti-mouse IgG antibody (BioLegend) or anti-mouse IgG1 antibody (SouthernBiotech). For IgE antibody detection, 96-well plates (3695, CORNING) were coated with 100 μg/mL OVA and biotin-conjugated anti-mouse IgE antibody (RME-1; BioLegend) was used at 1:500 dilution, followed by Streptavidin-HRP (R&D systems) at 1:200 dilution. TMB (tetramethylbenzidine) substrate or TMB High Sensitivity Substrate Solution (BioLegend) was then added to each well, and the plate was incubated for an additional 10 min; 2 N H_2_SO_4_ solution was added to stop the reaction. The absorbance at 450 nm was measured using a Multiskan FC microplate photometer (Thermo Fisher Scientific), and the basal absorbance at 570 nm was subtracted from the values.

### 2.11 Fluorescence immunohistostaining

Isolated LNs were fixed with 0.05% phosphate buffer containing 0.075 M L-lysine (pH 7.4), 0.01 M NaIO_4_, and 1% paraformaldehyde (PLP fixative) at 4 °C for 16–24 h (26). After fixation, LNs were equilibrated gradually with 10%, 20%, and 30% sucrose in PBS at 4°C, embedded in OCT compound (Sakura Finetech), and frozen at −80 °C. Frozen sections (10 µm) were prepared using a CM1860 cryostat (Leica Biosystems) and post-fixed with cold acetone for 3 min. Sections were stained with fluorochrome-conjugated antibodies and mounted with Permafluor Mountant (Thermo Fisher Scientific). Specimens were examined using an FV1200 confocal microscope (Olympus). Digital images were prepared using FV10-ASW (Olympus) and Adobe Photoshop (Adobe Systems).

### 2.12 Active cutaneous anaphylaxis reaction

The active cutaneous anaphylaxis assay was performed to detect indirectly the production of antigen-specific IgE with anaphylactic activity in the skin of the sensitized mice. Mice that have been subcutaneously inoculated with AoC or PBS were immunized with OVA (50 µg)/alum in the left hind footpad. After 28 days, the mice were challenged with 50 μg/10 µL OVA/PBS intradermally in the left ear. In the right ear, 10 μl of PBS was injected as a negative control. Ten minutes later, 200 µL of 1% Evans blue (Wako) in PBS was administered intravenously to the tail vein, and after 30 min, the mice were sacrificed, the auricles were photographed, and excised. The vascular leakage due to local anaphylaxis reaction was evaluated by extracting Evans blue dye from the auricle tissue soaking in formamide (Sigma) at 60 °C for 24 h and measuring the absorbance at 620 nm using a Multiskan FC microplate photometer (Thermo Fisher Scientific).

### 2.13 Statistical analysis

Microsoft Excel and GraphPad Prism 6 were used for the statistical analyses. The means of the two groups were compared using paired or unpaired Student’s *t*-tests. For comparing more than two groups, two-way ANOVA with Sidak’s multiple comparisons test was performed. Statistical significance was set at p < 0.05.

## 3 Results

### 3.1 Transdermal Ao entry induces hypertrophy and increased cell number in skin-draining LNs

**Figure 1:**
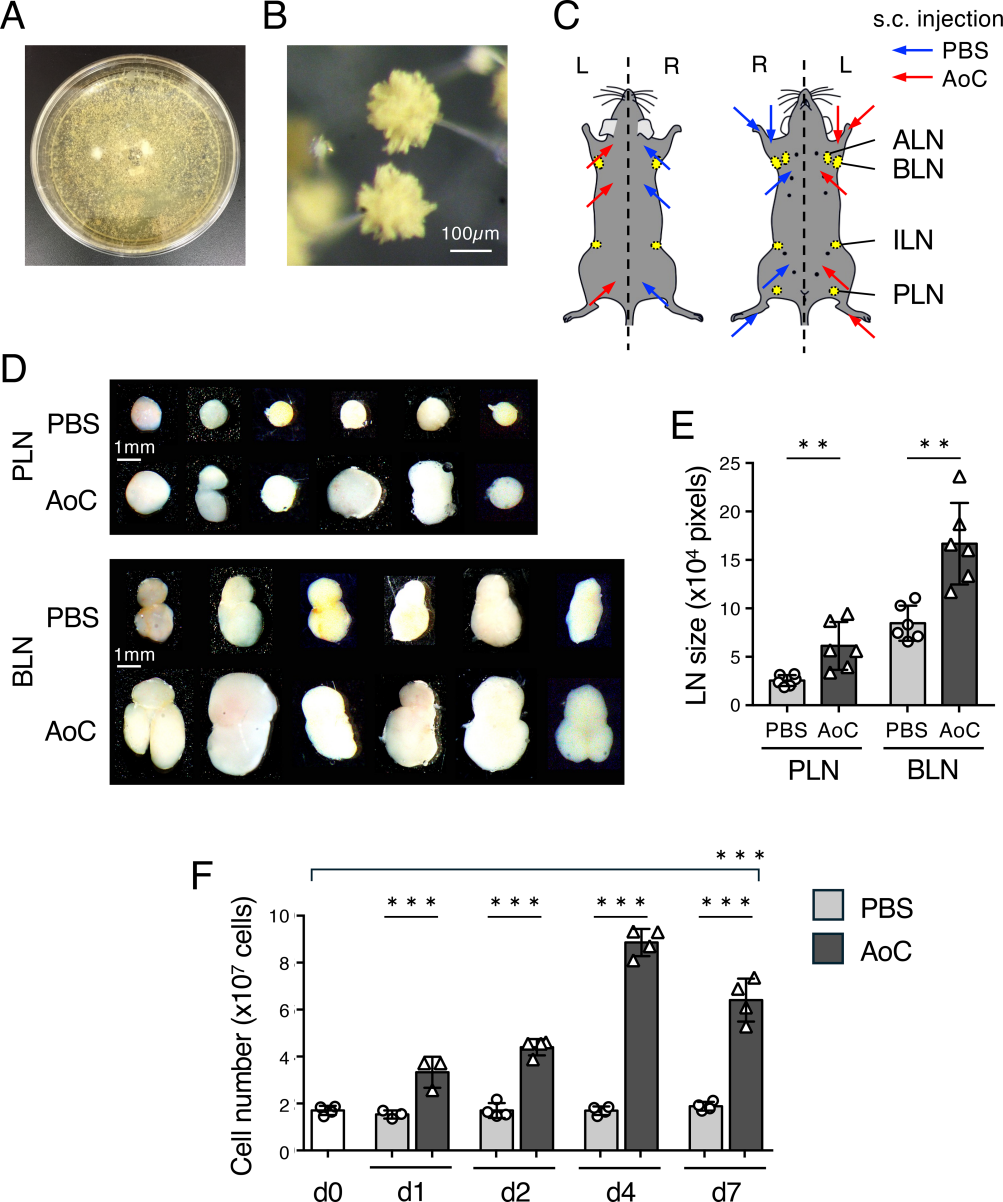
Subcutaneous AoC inoculation induces hypertrophy and increased cellularity in skin-draining LNs. **(A)** Ao culture on a Czapek-Dox agar plate. **(B)** Ao conidia clustered at the tip of conidiophores. **(C)** Schematic drawings of subcutaneous injection sites (PBS: blue arrows; AoC: red arrows) and LNs (yellow nodules) in a mouse. Dorsal (left) and ventral (right) views are shown. ALN, axillary LN; BLN, brachial LN; ILN, inguinal LN; PLN, popliteal LN. **(D)** Macroscopic views of PLNs and BLNs with or without AoC inoculation after 4 days. **(E)** Quantification of LN size. Each symbol indicates the measurement from a single LN. n = 6, Mean ± SD. Statistical analysis was performed using a paired *t*-test. **p < 0.01. **(F)** Time-course analysis of cell numbers in LNs following AoC inoculation. Total cell count from the four LNs on one side of the body is shown. d0: uninoculated steady-state LN. Each symbol indicates data from an individual mouse. n= 4, Mean ± SD. Statistical analysis was performed using two-way ANOVA (upper, ⊓) with Sidak’s post-test (lower, −). ***p < 0.001.

To investigate the immunological aspects induced in skin-draining LNs by transdermal Ao entry, the conidia (AoC) that were collected from solid Ao culture (**Figures 1A, B**; **Supplementary Figures 1A–C**) were inoculated subcutaneously at multiple sites on the left side of mice (with PBS injected contralaterally as a control), and superficial LNs were examined over time (**Figure 1C**). By 4 days after AoC inoculation, each LN had become hypertrophic to approximately twice its original size (**Figures 1D, E**). The total cell number of LNs showed a marked increase from day 1 post-inoculation, peaking on day 4 more than four-fold compared to that on the PBS-treated side, and then decreasing by day 7 (**Figure 1F**). No significant changes were observed in LNs on the PBS-injected contralateral side, indicating that a specific local response was induced by Ao. A fraction of the AoC inoculated into mice survived in the subcutaneous tissue for at least 7 days, while no live AoC were detected in the draining-LN even immediately after inoculation, suggesting that live AoC is rarely able to reach the LN (**Supplementary Figures 1G, H**). Therefore, transdermal Ao entry triggers a substantial early immune response in skin-draining LNs.

### 3.2 Ao induces B-cell-dominant increase and activation in LNs

**Figure 2:**
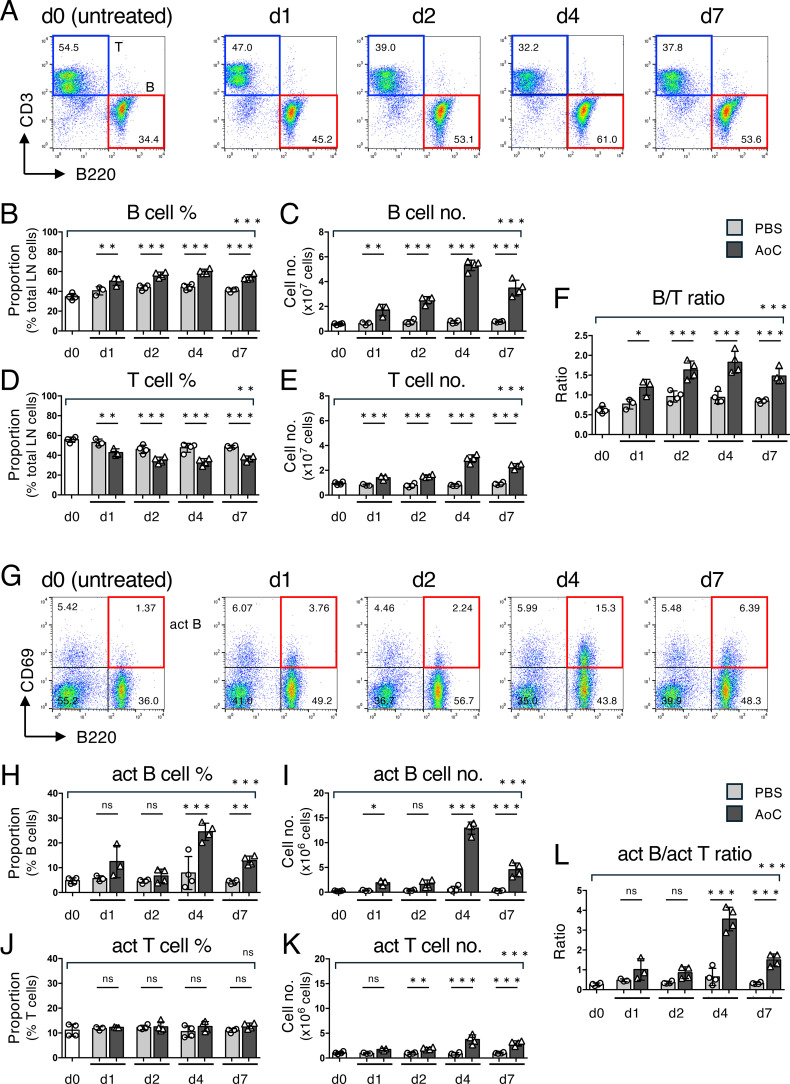
AoC induces B cell-dominant increase and activation in skin-draining LNs. **(A)** Flow cytometric analysis for determining the composition of B and T cells in LNs after AoC inoculation. Cells from LNs were isolated at the indicated time points after AoC or PBS injection and were stained for B220 and CD3. B cells and T cells were determined as B220^+^CD3^–^ (red) and CD3^+^B220^–^ (blue) gates, respectively. **(B–F)** Percentage changes **(B, D)** and numbers **(C, E)** of B and T cells, and the ratio of B cells to T cells **(F)** in LNs. Each symbol indicates data from an individual mouse. **(G)** Flow cytometric analysis for detecting activated B and T cells in LNs. Cells isolated from LNs at the indicated time points after AoC or PBS injection were stained for B220, CD3, and CD69. Activated (act) B and T cells were determined as B220^+^CD3^–^CD69^+^ (red) and CD3^+^B220^–^CD69^+^ (not shown) gates, respectively. **(H–L)** Percentage changes **(H, J)** and numbers **(I, K)** of act B and act T cells, and their ratio **(L)** in LNs. n= 4, Mean ± SD. Statistical analysis was performed using two-way ANOVA (⊓) with Sidak’s post-test (−). ns, not significant; *p < 0.05; **p < 0.01; ***p < 0.001.

To determine the detailed changes in immune cells in the LNs following AoC inoculation, we conducted flow cytometric analysis. Examination of lymphocyte subsets, that is, B cells and T cells, using B220 and CD3 as markers, respectively (**Figure 2A**), revealed that the proportion and number of B cells clearly increased starting 1 day after AoC inoculation, reaching a peak on day 4 (**Figures 2B, C**). In contrast, the proportion of T cells decreased, whereas their absolute number increased to some extent (**Figures 2D, E**); this change was weaker than that of B cells. Thus, the ratio of B to T cells markedly increased over time (**Figure 2F**).

Examining lymphocyte activation using CD69 expression (**Figure 2G**), B cells showed a marked increase in the CD69^+^ fraction on day 4 after AoC inoculation, which decreased by day 7 (**Figure 2H**). This was also evident from the increase in the number of activated cells (**Figure 2I**). Although a slight but statistically significant increase in CD69^+^ T cells was observed after day 4 (**Figures 2J, K**), it was not as pronounced as the increase in B cells. Thus, the ratio of activated B to T cells indicated a significant predominance of B cell activation (**Figure 2L**). These findings indicate that the transdermal entry of Ao induces a B-cell-dominant increase and activation in skin-draining LNs.

### 3.3 Ao induces several changes in dendritic cell subsets in LNs

**Figure 3:**
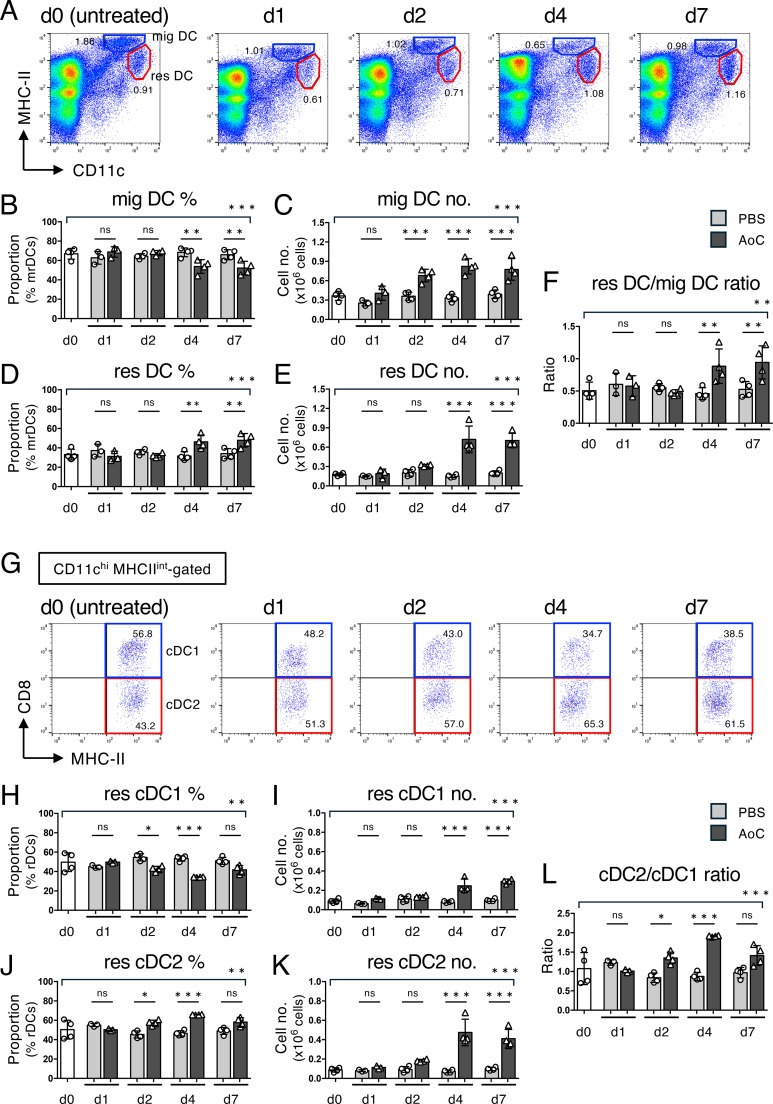
AoC induces the alterations of DC subsets in skin-draining LNs. **(A)** Flow cytometric analysis of migratory (mig) and resident (res) DC composition in LNs. Cells isolated from LNs at the indicated time points after AoC or PBS injection were stained for CD11c and MHC-II. mig DCs and res DCs were determined as CD11c^int^MHC-II^hi^ (blue) and CD11c^hi^MHC-II^int^ (red) gates. **(B–F)** Percentage changes in mig + res (mr) DCs **(B, D)** and numbers **(C, E)** of mig and res DCs, and the ratio of res DCs to mig DCs **(F)** in LNs. Each symbol indicates data from an individual mouse. **(G)** Flow cytometric analysis of cDC1 and cDC2 composition in the resident DC fraction. Cells isolated from LNs were stained for CD11c, MHC-II, and CD8. Resident cDC1 and cDC2 were determined as CD11c^hi^MHC-II^int^CD8^+^ (blue) and CD11c^hi^MHC-II^int^CD8^–^ (red) gates. **(H–L)** Percentage changes **(H, J)** and numbers **(I, K)** of resident cDC1 and cDC2, and their ratio **(L)**. n= 4, Mean ± SD. Statistical analysis was performed using two-way ANOVA (⊓) with Sidak’s post-test (−). ns, not significant; *p < 0.05; **p < 0.01; ***p < 0.001.

Various dendritic cell subsets are present in LNs and are involved in the induction and regulation of adaptive immunity (20, 27). Migratory dendritic cells (DCs) play a pivotal role in transporting antigens and pathogenic determinants from peripheral tissues to LNs, whereas subsets of resident DCs differentiate within LNs and have also been shown to participate in immune responses. Based on the expression of CD11c and MHC-II, we tracked the changes in these DC subsets in LNs over time following AoC inoculation (**Figure 3A**). On day 2, there was a slight increase in the proportion of migratory DCs; however, this change was not significant; from day 4 onward, the proportion decreased markedly (**Figures 3B, C**). Resident DCs showed the opposite trend, increasing significantly from day 4 (**Figures 3D, E**). Therefore, in terms of the resident/migratory ratio, resident DCs demonstrated significant dominance over migratory DCs from day 4 (**Figure 3F**).

We further subdivided resident DCs into cDC1 and cDC2 based on CD8 expression (**Figure 3G**) and found that cDC1 proportion transiently increased on day 1, significantly decreased from day 2 onward, and remained low thereafter (**Figures 3H, I**). In contrast, cDC2 followed the opposite trend, with its proportion increasing after day 2, and the ratio of cDC2 to cDC1 showed a significant cDC2 dominance (**Figures 3J–L**). Taken together, transdermal Ao entry induced substantial alterations in the composition of DC subsets in skin-draining LNs, particularly those characterized by a relative increase in resident cDC2.

### 3.4 Alterations in other immune cells

Although the increase and activation of T cells were not substantial, examination of CD4^+^ and CD8^+^ T cells (**Supplementary Figure 2A**) revealed a tendency for CD4^+^ T cells to become slightly dominant over CD8^+^ T cells from day 4 after AoC inoculation (**Supplementary Figures 2B–F**). The reason for this change is unclear; however, it is consistent with an increase in resident cDC2, which is closely associated with CD4^+^ T cells (28).

We also detected granulocytes and monocytes, which are generally mobilized from the blood during the early phase of inflammation, using CD11b and Ly6C (**Supplementary Figure 2G**). Both cell types increased most significantly in LNs at day 1 post-Ao inoculation but gradually decreased thereafter (**Supplementary Figures 2H–K**). This suggests that Ao can induce an early inflammatory response in skin-draining LNs, leading to the mobilization of inflammatory leukocytes from the circulation.

### 3.5 The response to Ao is partially reduced by heat inactivation

Thus far, we have demonstrated that live AoC induces a remarkable LN response. However, it is assumed that most of the Ao present in fermented foods or products is inactivated by heat or other treatments. We examined the immunostimulatory activity of AoC, which loses its proliferative ability after heat treatment. In preliminary examinations, the growth potential of AoC after treatment at various temperatures showed a complete loss at 70 °C for 10 min (**Supplementary Figure 1D**). This heat-inactivated AoC was subcutaneously administered, and changes in the draining LNs 4 days later were compared with those of the live AoC. Consequently, although heat-inactivated AoC induced LN enlargement with increased cell number, the activity was significantly reduced compared with that of live AoC (**Supplementary Figure 3A**). This reduction was also observed with an increase in the number and proportion of B cells (**Supplementary Figures 3B–D**). Furthermore, although a slight increase in activated B cells was observed with heat-inactivated AoC, this change was not significant compared with the control PBS (**Supplementary Figures 3E, F**), suggesting that heat inactivation virtually eliminated the early B cell stimulatory activity of AoC. Heat-inactivated AoC induced a reduction in migratory DCs and an increase in resident DCs, although the extent of the change was weaker than that of live AoC (**Supplementary Figures 3G, H**). A substantial increase in cDC2 within resident DCs was also observed, with changes slightly weaker to those in live AoC (**Supplementary Figures 3I, J**). Therefore, inactive AoC retains some activity to induce LN responses; however, that is markedly reduced compared with live AoC.

### 3.6 The LN response induced by Ao is partially recapitulated by β-glucan

**Figure 4:**
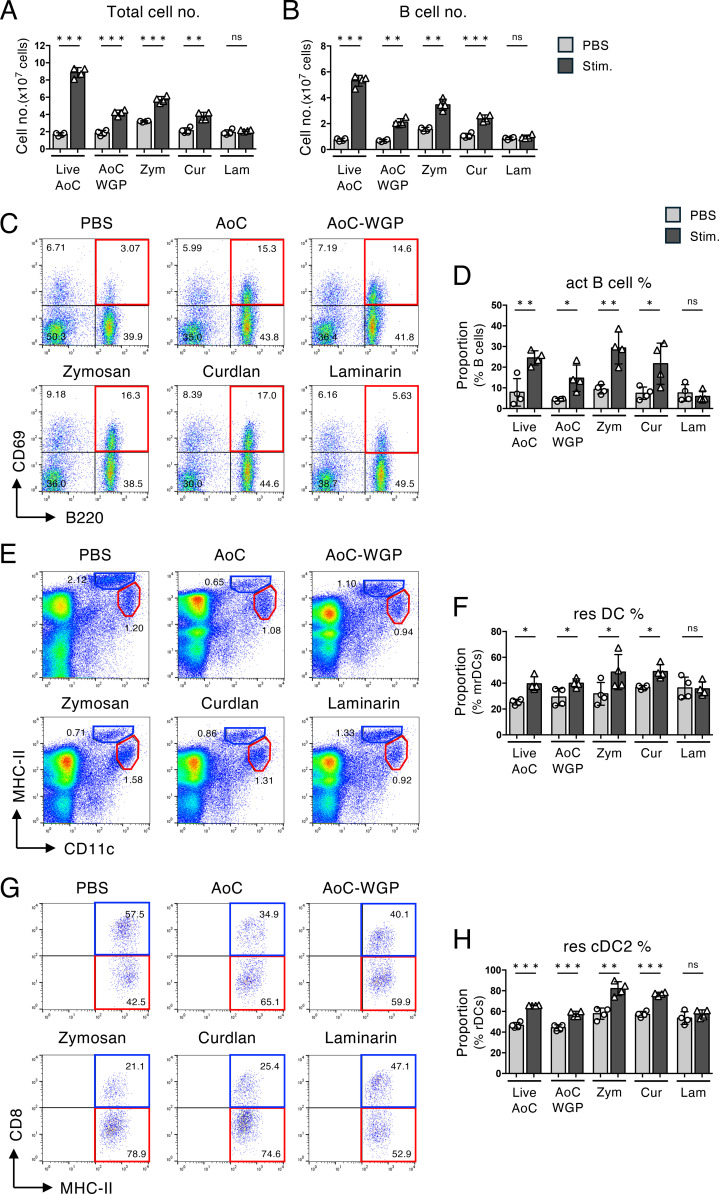
β-glucans partially induce LN changes. **(A, B)** Total cells **(A)** and B cells **(B)** in skin-draining LNs following the inoculation of live AoC, AoC-WGP, zymosan (Zym), curdlan (Cur), and laminarin (Lam). **(C–H)** Flow cytometric analysis for the composition of activated B cells **(C, D)**, res DCs **(E, F)**, and res cDC2 **(G, H)** with the indicated substances. n= 4, Mean ± SD. Statistical analysis was performed using a paired *t*-test. ns, not significant; *p < 0.05; **p < 0.01; ***p < 0.001.

β-glucans are the main component of fungal cell wall, including those of Ao, and have long been reported to exhibit immunostimulatory activities (29–31). Therefore, we prepared whole-glucan particle (WGP) by treating AoC with acid and alkali to concentrate the cell wall components (**Supplementary Figures 1E, F**). We then subcutaneously inoculated AoC-WGP, zymosan (a crude yeast cell wall extract), curdlan (a linear β-glucan derived from bacteria), or laminarin (a low-molecular-weight β-glucan derived from seaweed), and compared LN changes to those observed with live AoC (**Figure 4**). Note that AoC-WGP and β-glucans were assessed on day 2, when the most intensive LN response was confirmed to occur, whereas live AoC was compared with the response on day 4. AoC-WGP, zymosan, and curdlan elicited responses equivalent to or slightly weaker than those elicited by live Ao, including increased total LN cell numbers, elevated B cell numbers and activation, and altered proportions of DC subsets (**Figure 4**). In contrast, laminarin did not induce this response. These findings suggest that the LN responses induced by Ao are caused in part by particulate or high molecular weight β-glucans.

### 3.7 Cells expressing Dectin-1 in LNs and its downregulation by Ao

Dectin-1 functions as a β-glucan receptor (32, 33), and the above results are consistent with the fact that laminarin can bind to Dectin-1 but does not transmit signals (34). Detection of Dectin-1 expression in various immune cells of the mouse LN revealed that lymphocytes, such as B cells and T cells, showed almost no surface expression, whereas clear expression was detected in myeloid cells, including DCs and macrophage subsets, monocytes, and granulocytes (**Supplementary Figures 4A–E**). Additionally, a search of RNA-sequence analysis data in the ImmGen database (http://rstats.immgen.org/Skyline/skyline.html), which includes expression data for equivalent cell types in the spleen, confirmed similarly high Dectin-1 (Clec7a) expression in myeloid lineages (**Supplementary Figure 4F**). Therefore, β-glucan derived from Ao is most likely to stimulate myeloid cells within LNs and induce subsequent responses.

Following subcutaneous AoC inoculation, we examined alterations in Dectin-1 expression in migratory and resident DCs in LNs over time. We anticipated that DC activation might simply increase Dectin-1 expression, or that β-glucan binding might induce Dectin-1 internalization, leading to the reduction of its cell surface expression. While no significant changes were observed in the PBS-injected control side, a marked Dectin-1 reduction in migratory DCs was detected on the AoC-injected side and was most pronounced on day 2 (**Supplementary Figures 5A, B**). In resident DCs, a decreasing trend was also observed at both 1–2 days post-injection, with no significant difference compared to PBS; a significant decrease was detected at 4 days post-injection (**Supplementary Figures 5A, B**). Therefore, these suggest that AoC-derived β-glucan transduces signal via Dectin-1 and promotes its internalization, leading to decreased expression on the cell surface (35, 36). Since migratory DCs are presumed to come into first contact with β-glucan in subcutaneous tissue, Dectin-1 downregulation is suggested to be induced more strongly from an early stage.

### 3.8 Ao changes some cytokine expressions in LNs

**Figure 5:**
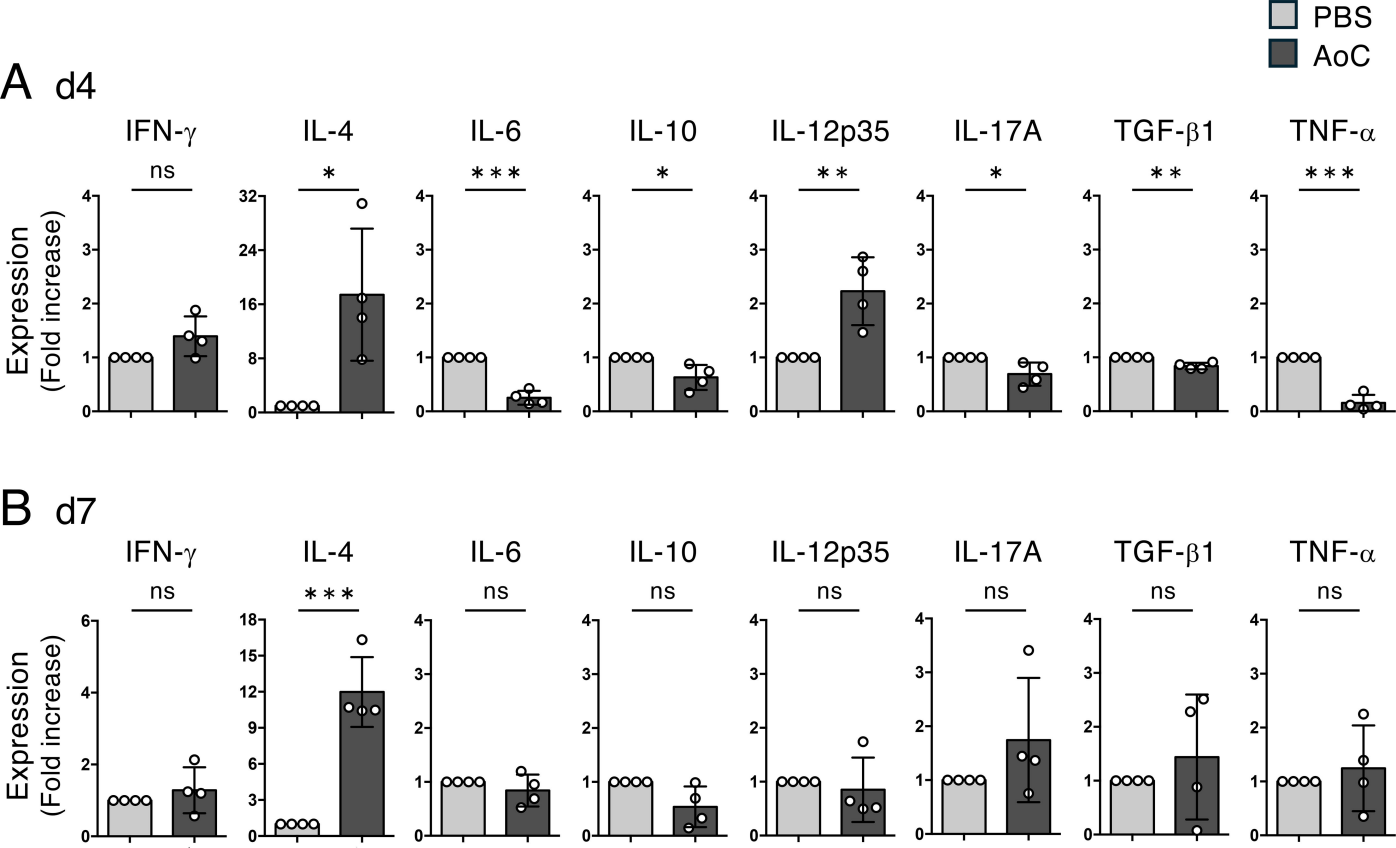
AoC-induced changes of cytokine expression in skin-draining LNs. **(A, B)** Quantitative RT-PCR analysis for the expression of cytokines in LNs after 4 days **(A)** and 7 days **(B)** of PBS (control) or AoC subcutaneous inoculation. Expression levels of cytokine genes were normalized to that of GAPDH and shown as fold increase relative to that in the PBS injected side of LNs. Each symbol indicates data from an individual mouse. n= 4, Mean ± SD. Statistical analysis was performed using an unpaired *t*-test. ns, not significant; *p < 0.05; **p < 0.01; ***p < 0.001.

Given that AoC induces a variety of alterations in immune cells within LNs, we examined changes in cytokine expression as an indicator of the type of ongoing response. On days 4 and 7 after AoC inoculation, RNA was extracted from whole LN cells, and quantitative RT-PCR analysis was performed to evaluate the changes in expression levels on the AoC-injected side of the LNs relative to the PBS-injected control side. The most prominent change observed on day 4 was a marked increase in IL-4 expression (**Figure 5A**). In addition, IL-12 exhibited a significant increase. However, IFN-γ showed no significant change, while IL-6, IL-10, IL-17, TGF-β, and TNF-α demonstrated a clear decrease. This was an unexpected result considering the marked swelling and various immune cell alterations in the LNs on day 4 after AoC inoculation. Furthermore, IL-4 continued to show a marked increase in expression level even on day 7, whereas all other factors showed no significant differences compared to the control, suggesting that they had returned to nearly steady-state levels (**Figure 5B**).

We also examined cytokines production by T cells in LNs 7 days after AoC inoculation using intracellular staining and flow cytometry. The results showed that IL-4 production in CD4^+^ T cells tended to be slightly higher, but without significant difference, in AoC inoculation compared to the PBS-injected side (**Supplementary Figure 6B**). Moreover, no significant changes were observed in the production of IFN-γ, IL-17, TNF-α, and IL-10 (**Supplementary Figures 6A, C, D, E**). In addition, the proportion of FoxP3^+^ regulatory T cells did not show any significant changes as well (**Supplementary Figure 6F**). On the other hand, a slight but significant increase of IFN-γ production in CD8^+^ T cells was observed following AoC inoculation (**Supplementary Figure 6G**). Therefore, despite significant changes in LN cellular composition, AoC inoculation did not induce a robust response; rather a situation in which overall cytokine expression was restrained. Among these, only IL-4 showed a marked increase in expression throughout the LNs.

### 3.9 Ao inoculation induces Ao-specific antibody production

**Figure 6:**
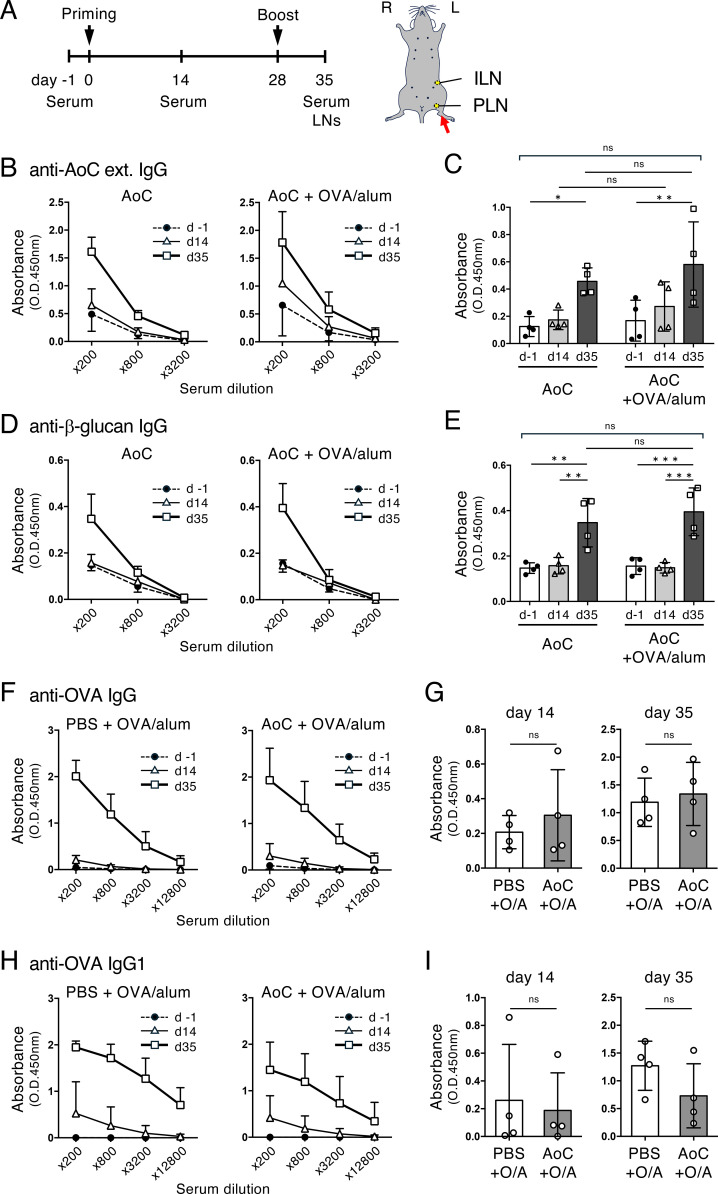
AoC induces specific antibodies against AoC proteins and β-glucan. **(A)** Scheme of immunization and serum collection. Heat inactivated AoC and/or OVA/alum were subcutaneously injected to the left hind footpad (red arrow) on day 0 (priming) and day 28 (boost). Sera were collected at days -1, 14, and 35. **(B, C)** Detection of anti-AoC IgG antibody production in sera. Sera from immunized mice (n = 4 each) at indicated time points were serially diluted and analyzed using ELISA for the antibody specific to AoC protein extract. Data are shown as the absorbance (OD) at 450 nm. To compare the results of AoC alone and AoC+OVA/alum, the serum dilution points at ×800 are also shown in **(C)**. **(D, E)** Detection of anti-β-glucan IgG antibody production in sera. To compare the results of AoC alone and AoC+OVA/alum, the serum dilution points at ×200 are also shown in **(E)**. Each symbol indicates the data from an individual mouse. n= 4, Mean ± SD. Statistical analysis was performed using two-way ANOVA (⊓) with Sidak’s post-test (−). ns, not significant; *p < 0.05; **p < 0.01; ***p < 0.001. **(F, G)** Detection of anti-OVA IgG antibody production in sera. To compare the results of OVA/alum with or without AoC, the serum dilution points at ×200 (day 14) and ×800 (day 35) are shown in **(G)**. **(H, I)** Detection of anti-OVA IgG1 antibody production in sera. To compare the results of OVA/alum with or without AoC, the serum dilution points at ×800 (day 14) and ×3200 (day 35) are shown in **(I)**. Each symbol indicates the data from an individual mouse. n= 4, Mean ± SD. Statistical analysis was performed using an unpaired *t*-test. ns, not significant.

To address whether Ao induces antigen-specific adaptive immunity leading to antibody production, AoC was inoculated alone or in combination with OVA with an alum adjuvant as a vaccination mimetic (**Figure 6A**). Serum was collected 14 days after the primary inoculation and again 7 days (35 days) after the secondary booster challenge 4 weeks later. Antigen-specific antibody production in sera was evaluated using ELISA. IgG antibodies reacting with AoC protein extracts were detected at both 14 and 35 days after AoC treatment alone or in the presence of OVA/alum, showing an increasing trend over time (**Figures 6B, C**). However, the alum adjuvant showed little enhancement in antibody production. IgG antibody against β-glucan (curdlan) was also detected, albeit at a weaker level (**Figures 6D, E**). In addition, total IgG and IgG1 specific to OVA did not change with or without AoC (**Figures 6F–I**), suggesting that Ao had little additional impact on the response to other antigens/adjuvants. Using flow cytometry analysis, we observed a clear increase in the proportion of IgG1^+^ fraction in B cells on day 7 after re-challenge with AoC, whereas no increase was observed in the memory B cell fraction (**Supplementary Figure 7**). Accordingly, although antigen-specific antibody production is detectable, this suggests that AoC alone does not elicit a robust memory response.

**Figure 7:**
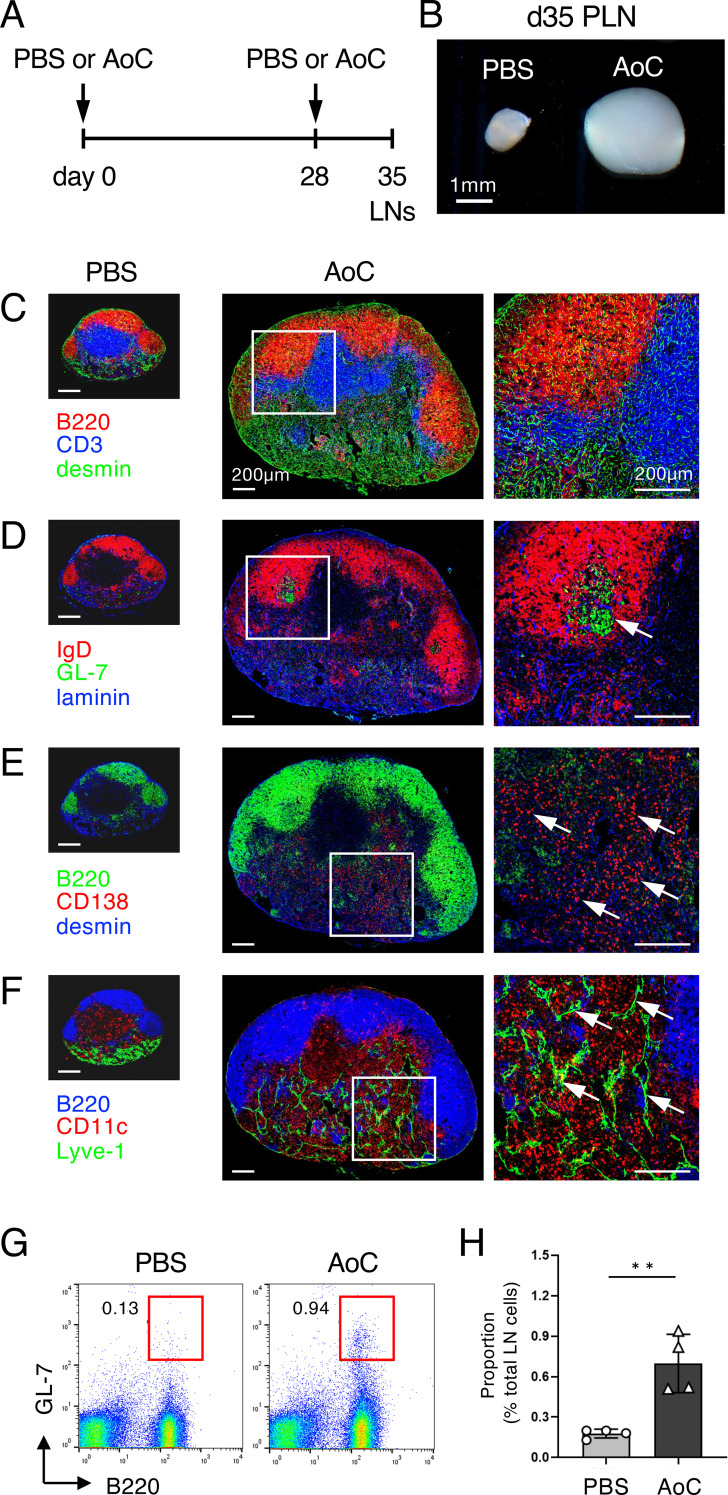
AoC induces dynamic LN tissue reorganization. **(A)** AoC or PBS inoculation scheme. **(B)** Macroscopic views of popliteal LNs excised from mice 35 days after PBS or AoC inoculation. **(C–F)** Fluorescent immunostaining of LNs 35 days after PBS or AoC inoculation. Tissue sections were stained for the indicated markers and examined using confocal microscopy. Boxed regions are magnified in the right panels. Images are the representatives of more than three LNs from three mice in each treatment. Arrows indicate a germinal center **(D)**, plasma cells **(E)**, and the medullary lymphatic sinuses **(F)**. **(G, H)** Flow cytometric analysis for detecting germinal center B cells (B220^+^GL7^+^ cells) in skin-draining LNs upon AoC inoculation. n = 4, Mean ± SD. Statistical analysis was performed using a paired *t*-test. **p < 0.01.

Fluorescent antibody staining of AoC-sensitized and -boosted LN tissue sections revealed hypertrophy with a marked increase in lymphocyte compartments, particularly a dramatic enlargement of B cell follicles (**Figures 7A–C**). Within the follicles, germinal centers were often formed, reflecting the active proliferation of antibody-producing B cells, whereas numerous plasma cells accumulated in the medullary region (**Figures 7D, E**). In addition, it was characterized by a marked expansion of the medullary region with reticular reorganization in sinuses composed of LYVE-1^+^ lymphatic endothelial cells (**Figure 7F**). These findings are consistent in part with flow cytometric observations showing a significant increase in B220^+^GL7^+^ germinal center B cells in LNs following a similar AoC administration. (**Figures 7G, H**). Therefore, Ao stimulation alone can reorganize the tissue structure of the LNs and induce an environment that supports antibody production.

### 3.10 Ao suppresses allergic responses

**Figure 8:**
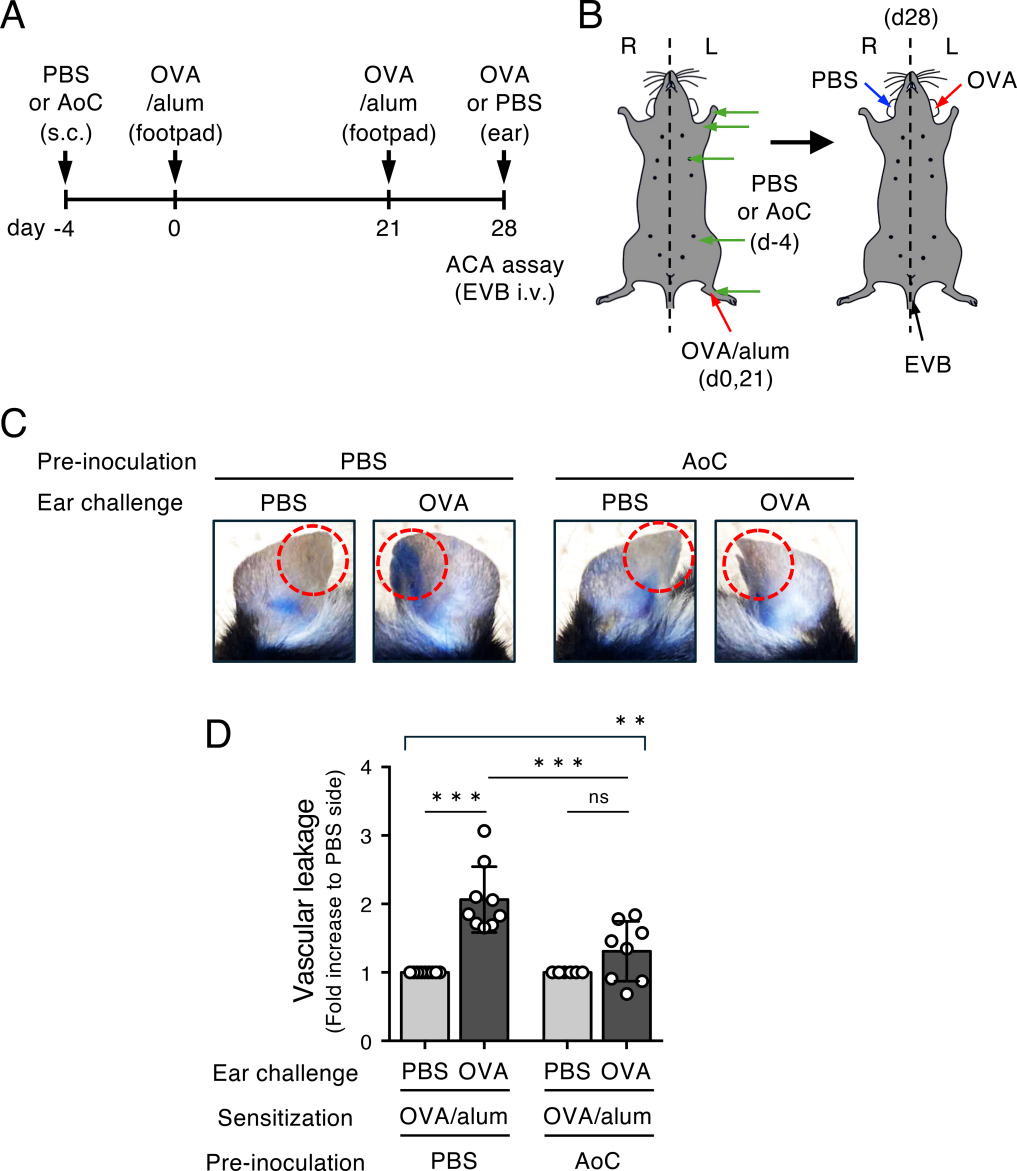
AoC pre-inoculation inhibits allergic response. **(A, B)** Experimental procedure for the acute cutaneous anaphylaxis (ACA) assay. Heat-inactivated AoC or PBS were subcutaneously injected on day -4 and OVA/alum was injected to the left hind footpad on days 0 and 21. On day 28, OVA and PBS were injected to the left and right ears, respectively, for the active cutaneous anaphylaxis (ACA) assay to detect vascular leakage of intravenously-injected Evans blue (EVB) dye. **(C)** Photographs of the ears after inducing local anaphylaxis reaction. Dotted circles indicate the site of OVA or PBS (stim.) injections. Representatives from each group (pre-inoculation with PBS: n= 9 and AoC: n= 8) are shown. **(D)** Vascular leakage on ACA reaction. EVB dye was extracted from the excised auricles, and its absorbance was measured at 620 nm. Data are shown as the fold increase relative to that of the PBS (control) side. Each symbol indicates data from an individual mouse. Pre-inoculation with PBS: n= 9 and AoC: n= 8, Mean ± SD. Statistical analysis was performed using two-way ANOVA (⊓) with Sidak’s post-test (−). ns, not significant; **p < 0.01; ***p < 0.001.

Ao inoculation markedly increased IL-4 expression in LNs but tended to decrease or unalter other cytokines, whereas it did not promote OVA-specific IgG antibody production. This prompted us to speculate that Ao might influence the induction of some allergic responses. To test this, we examined the effect of prior AoC inoculation on increased vascular permeability in an antigen-specific skin anaphylactic reaction as an indicator of an IgE-dependent allergic response (**Figures 8A, B**). AoC or PBS was preinjected subcutaneously at multiple sites, followed by sensitization with OVA/alum. A second OVA/alum challenge was performed on day 21, and 1 week later, the OVA-specific anaphylactic reaction in the auricle was evaluated by assessing the vascular permeability of Evans blue dye leakage from the blood. In the control group without Ao inoculation, skin anaphylactic reactions were induced by increased vascular permeability in an antigen-dependent manner (**Figures 8C, D**). In contrast, the AoC-pretreated group showed a significant decrease in vascular leakage following OVA administration. Although we were unable to detect OVA-specific IgE antibodies in the blood under these settings, the findings above suggest that subcutaneous AoC inoculation restrained the anaphylactic reaction by some mechanism.

**Figure 9:**
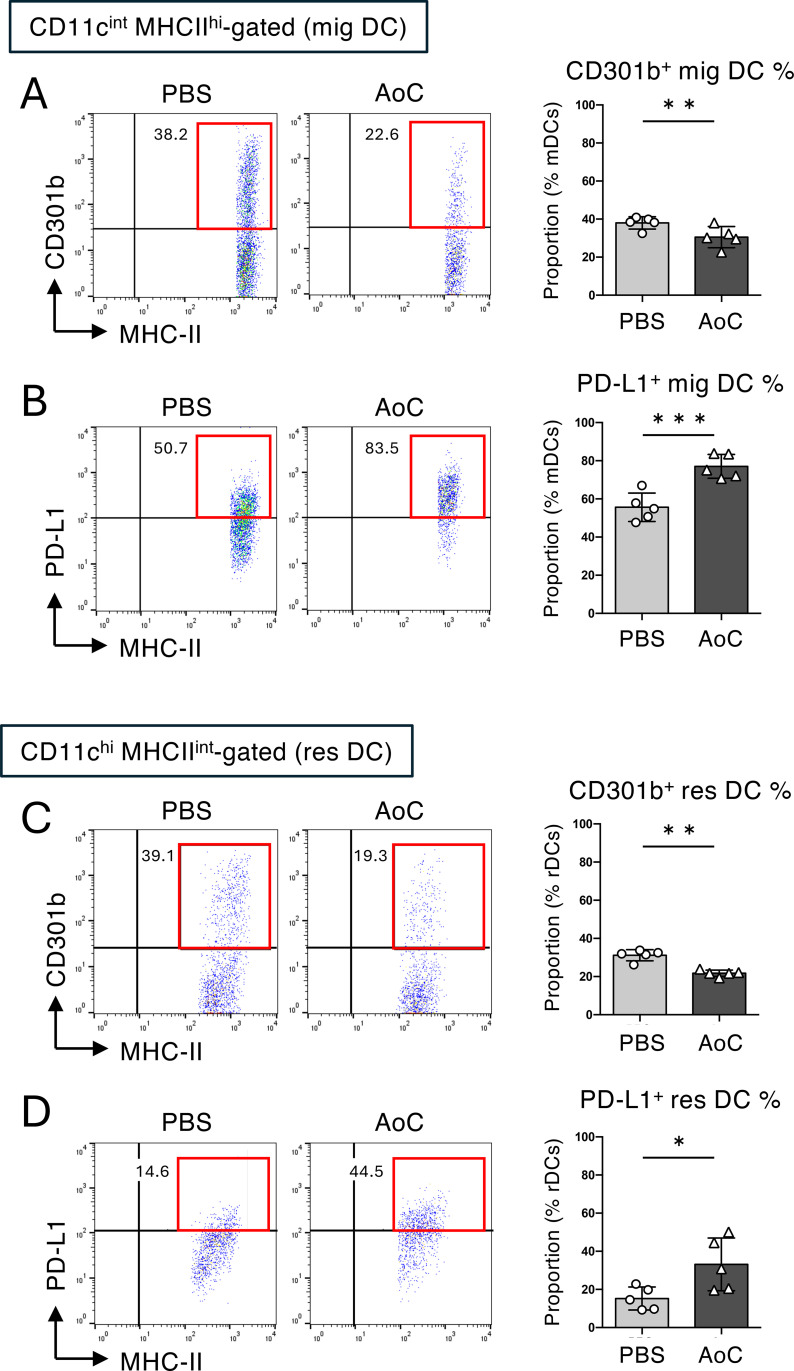
AoC induces a regulatory property of DC subsets in skin-draining LNs. Flow cytometric analysis for detecting CD301b and PD-L1 expression in migratory (mig) DCs **(A, B)** and resident (res) DCs **(C, D)**. Cells isolated from LNs 4 days after AoC or PBS inoculation were stained for CD11c, MHC-II, and CD301b or PD-L1. mig DCs and res DCs were determined as CD11c^int^MHC-II^hi^ and CD11c^hi^MHC-II^int^, respectively. n = 5, Mean ± SD. Statistical analysis was performed using a paired *t*-test. *p < 0.05; **p < 0.01; ***p < 0.001.

In separate observations, we found that the proportion of the CD301b^+^ DC fraction—known to play a key role in inducing allergic responses (37)—clearly decreased in both migratory and resident DCs in LNs 4 days after AoC inoculation, while PD-L1 expression, which confers immunosuppressive functions (38, 39), increased significantly (**Figures 9A–D**). The findings suggest that these changes in DC subsets may exert an overall inhibitory effect on the induction of early responses, including allergic reaction. Consequently, transdermal Ao entry has been suggested to play an immunomodulatory role in the skin-draining LNs, ultimately inhibiting allergic responses.

## 4 Discussion

In this study, we demonstrated that transdermal entry of the non-pathogenic fermentative fungus Ao induces remarkable responses in skin-draining LNs within a week, that is, tissue hypertrophy along with various alterations in immune cells. The clear immune response ultimately led to Ao antigen-specific antibody production. However, the Ao-induced LN response is not particularly intense; instead, it exerts an inhibitory effect on allergic responses. In the early phase following Ao inoculation, an inflammatory response, primarily by granulocyte and monocyte mobilization, was evident. Subsequently, the increase and activation of B cells became prominent within several days, peaking on day 4, followed by an increase in resident cDC2, which was a characteristic aspect. Although a slight increase in T cell activation was detected, it did not appear to constitute a strong response. This is thought to arise from the fact that, while B cells can be activated by various stimuli in an antigen-dependent and -independent manners (40, 41), T cell activation is strictly regulated by specific antigen recognition.

A similar early LN response was partially reproduced, albeit attenuated, by inoculation with heat-inactivated AoC, suggesting that it was caused by the combined action of the heat-resistant and heat-sensitive components. This also implies that Ao that has undergone thermal denaturation through some treatments or cooking, retains its immunostimulatory activity, whereas the live state exhibits a higher ability to stimulate the immune system. Moreover, since many of the LN responses to live AoC can also be reproduced by high molecular weight β-glucans and AoC-WGP, β-glucan derived from Ao is assumed to be the primary stimulatory component. However, while the peak response to live AoC occurs on day 4 post-inoculation, that to β-glucans is observed as early as day 2, suggesting that live conidia require more time for releasing β-glucans due to their inner cell wall destruction (42). The released β-glucan is presumed to stimulate myeloid cells such as DCs and macrophages via Dectin-1, thereby promoting the secretion of inflammatory mediators and cytokines; this in turn possibly leads to B cell activation, increasing their numbers. Significant B-cell activation accompanied by CD69 induction was also evident; CD69 expression is known to suppress lymphocyte egress from lymphoid tissues (43, 44), indicating that it retains B cells in LNs and leads to an increase in cell number. Moreover, live AoC induced the strongest increase in lymphocytes in LNs than purified β-glucans, suggesting that immunostimulatory components other than β-glucans could also be involved in this phenomenon.

The compositional changes in the DC subsets observed in the LNs induced by AoC were noteworthy. Upon pathogenic stimulation, numerous DCs migrating from peripheral tissues are expected to reach LNs within a few days, thereby transiently increasing their proportion (45–47). However, this trend was not substantial for AoC inoculation, and a decrease in the proportion of migratory DCs was observed from day 4 onward. Consequently, while both migratory and resident DCs showed an increasing trend in cell numbers, resident DCs ultimately became predominant. The marked decrease of surface Dectin-1 expression observed on migratory DCs indicates that AoC-derived β-glucan in fact stimulates these cells (35, 36). It is unclear whether the relatively modest increase in migratory DCs in the early phase in LNs is directly related to weak T cell activation, but a mild or restricted LN response to AoC is consistent with these findings. The clear shift to cDC2 dominance within resident DCs was reproducible. Because cDC2 is known to be a DC subset responsible for antigen presentation to CD4^+^ T cells (20, 28, 48), this is consistent with the fact that the T cell subset becomes dominated by CD4^+^ T cells by day 7.

AoC alone inoculation or vaccine-mimetic immunization with other foreign antigen (OVA) and an adjuvant revealed that it finally induces antibody production specific to AoC antigens and β-glucan. Moreover, antibody titers further increased after booster inoculation, indicating the acquisition of immune memory against Ao. However, since the alum adjuvant did not significantly increase antibody production compared to AoC inoculation alone, AoC already possessed some adjuvant activity, and the addition of alum did not enhance this effect. In addition, no significant enhancement of antibodies against other antigens was observed with or without AoC, suggesting that AoC does not possess additive stimulatory activity to enhance the response to OVA/alum. This overall mild antibody production may indicate that the initial response to Ao is not practically intense. In fact, while AoC inoculation alone slightly increased IgG1^+^ B cells that had undergone class switching, it did not significantly increase memory B cell fraction.

AoC inoculation markedly increased IL-4 expression in LNs. However, many other cytokines showed decreased or unchanged expression on day 4. In particular, the lack of an increase in inflammatory cytokines suggests that the response was somehow restrained. The transient increase of IL-12, which can induce a type 1 response, on day 4 is considered to act as a counter inhibitor to the promotion of a type 2 response by IL-4 (49, 50). Regarding cytokine production by CD4^+^ T cells, although there was a tendency toward increased IL-4, this was not statistically significant, and the levels of other cytokines did not change following AoC inoculation. The only exception was a slight increase in IFN-γ production in CD8^+^ T cells, which may be consistent with a tendency toward suppression of the type 2 response. A closely related finding is that pre-injection of AoC suppresses antigen-specific anaphylaxis likely mediated by IgE as an allergic response. This suggests that prior contact with Ao inhibits the induction of allergies. We initially assumed that AoC inoculation might enhance allergic responses given its ability to increase IL-4 expression, which is a potent inducer of IgE production. However, the suppression of anaphylaxis suggests the induction of a regulatory trend that overrides pro-allergic IL-4 function. We finally found that AoC inoculation led to a decrease in the CD301b^+^ fraction and an increase in PD-L1 expression in LN DCs, which might be responsible for or contribute to such an intriguing regulatory response. Altogether, transdermal exposure to Ao may play a role in controlling the balance between type 1 and type 2 responses in the LNs.

The relevance between exposure to microorganisms or pathogens and the suppression of immune-related diseases, such as allergies, is known as the “hygiene hypothesis” or “old friends hypothesis” (2–5). It is believed that contact with diverse microorganisms, particularly during infancy, is crucial for proper development and control of the immune system. The inhibition of allergic responses in adult mice following sensitization to Ao suggests that immune responses may be appropriately modulated, even after growth, via contact with non-pathogenic microorganisms. Proactive contact with harmless fermentative microorganisms, such as Ao, may be a promising countermeasure to immune dysfunction in modern society, owing to reduced microorganisms in the surroundings.

Taken together, our findings shed light on some aspects of the immune response occurring in skin-draining LNs to the transdermal entry of the non-pathogenic fungus Ao, which had previously been largely unknown. Ao is a useful microorganism for humans because of its fermentation capabilities; moreover, it holds promise for applications in regulating immune system balance. It is necessary to further elucidate the specific types of immune responses elicited by Ao, the nature of the regulatory responses, the relationship with immune memory, and the mechanism for controlling allergic responses, thereby deepening our understanding of the interactions between this unique microorganism and animal physiology.

## Data Availability

The datasets presented in this study can be found in online repositories. The names of the repository/repositories and accession number(s) can be found in the article/**Supplementary Material**.
