# Supplementary material for: Transdermal entry of a non-pathogenic filamentous fungus Aspergillus oryzae induces an immunomodulatory response in skin-draining lymph nodes
Source: Front Immunol. 2026 Jun 15;17:1815568. doi: 10.3389/fimmu.2026.1815568 (PMC13310732; doi:10.3389/fimmu.2026.1815568)
Supplement: Supplementary file 1 [file DataSheet1.pdf]

# Supplementary Figure 1

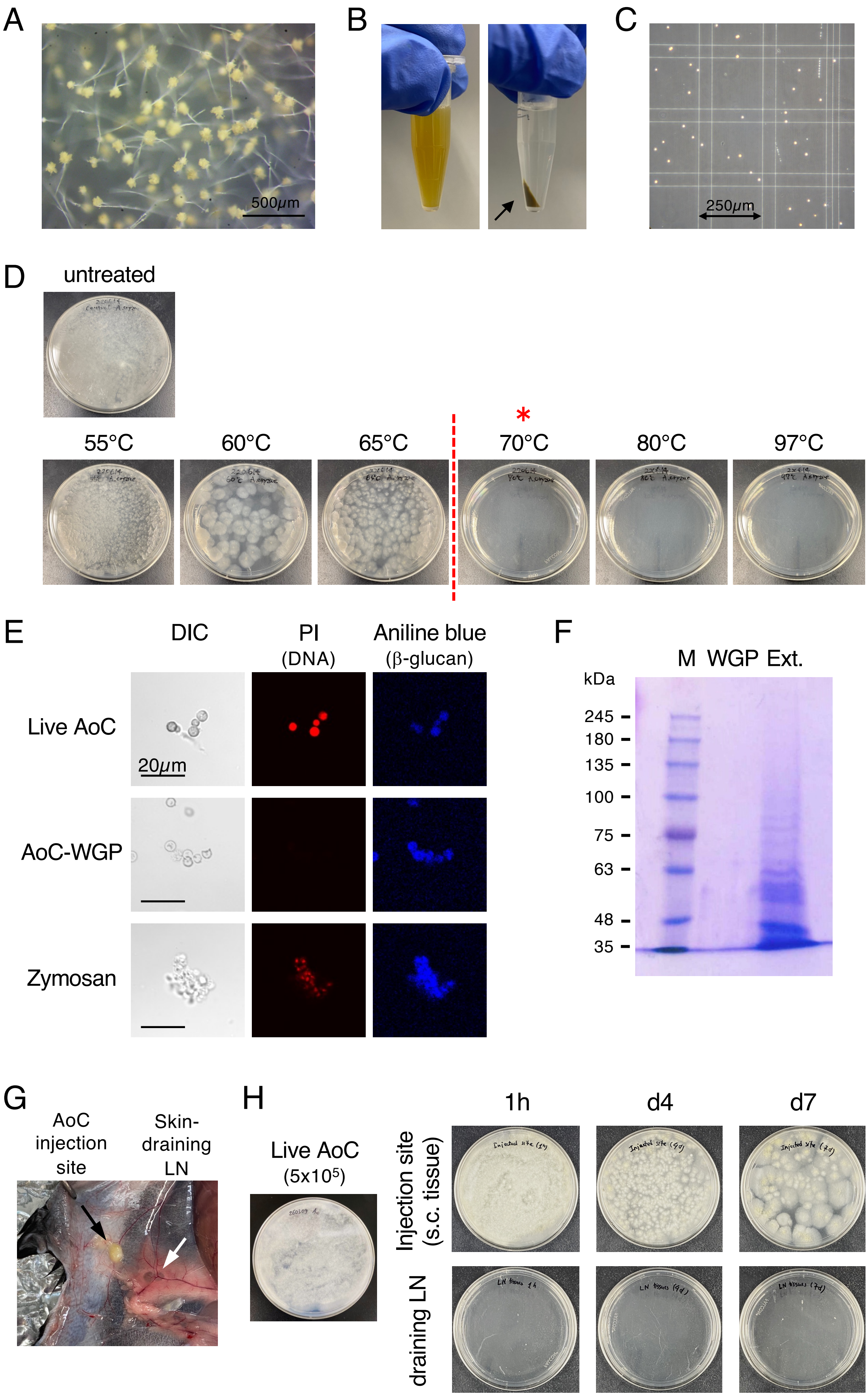

**Supplementary Figure 1.** Preparation of live AoC, heat-inactivated AoC, and AoC-WGP.

**(A)** Ao cultured on Czapek-Dox agar forming dense conidiophores. **(B)** Suspension of AoC collected from the culture (left) and the pellet precipitated using centrifugation (right, arrow). **(C)** Enumeration of AoC using a hemocytometer. (**D)** Determination of the temperature at which growth capacity is completely lost owing to heat treatment. AoC ($1 \times 10^6$ cells) treated at each temperature for 10 minutes were inoculated onto Czapek-Dox agar plates and incubated for 3 days. **(E)** Confocal examination of live AoC, AoC-WGP, and zymosan stained with PI and aniline blue. DIC, differential interference contrast. **(F)** SDS-PAGE (10%) was performed to confirm protein removal in AoC-WGP and protein extraction from AoC (Ext.). M: size marker. **(G)** Survival of AoC after in vivo inoculation. Appearance of AoC-inoculated subcutaneous tissue (black arrow) and regional skin-draining LN (white arrow) in mouse. **(H)** Evaluation based on the growth of surviving AoC remaining in subcutaneous tissues and draining LNs 1 hour, 4 days, and 7 days after inoculation. The tissue homogenate was seeded onto Czapek-Dox agar plates and cultured for 4 days.

# Supplementary Figure 2

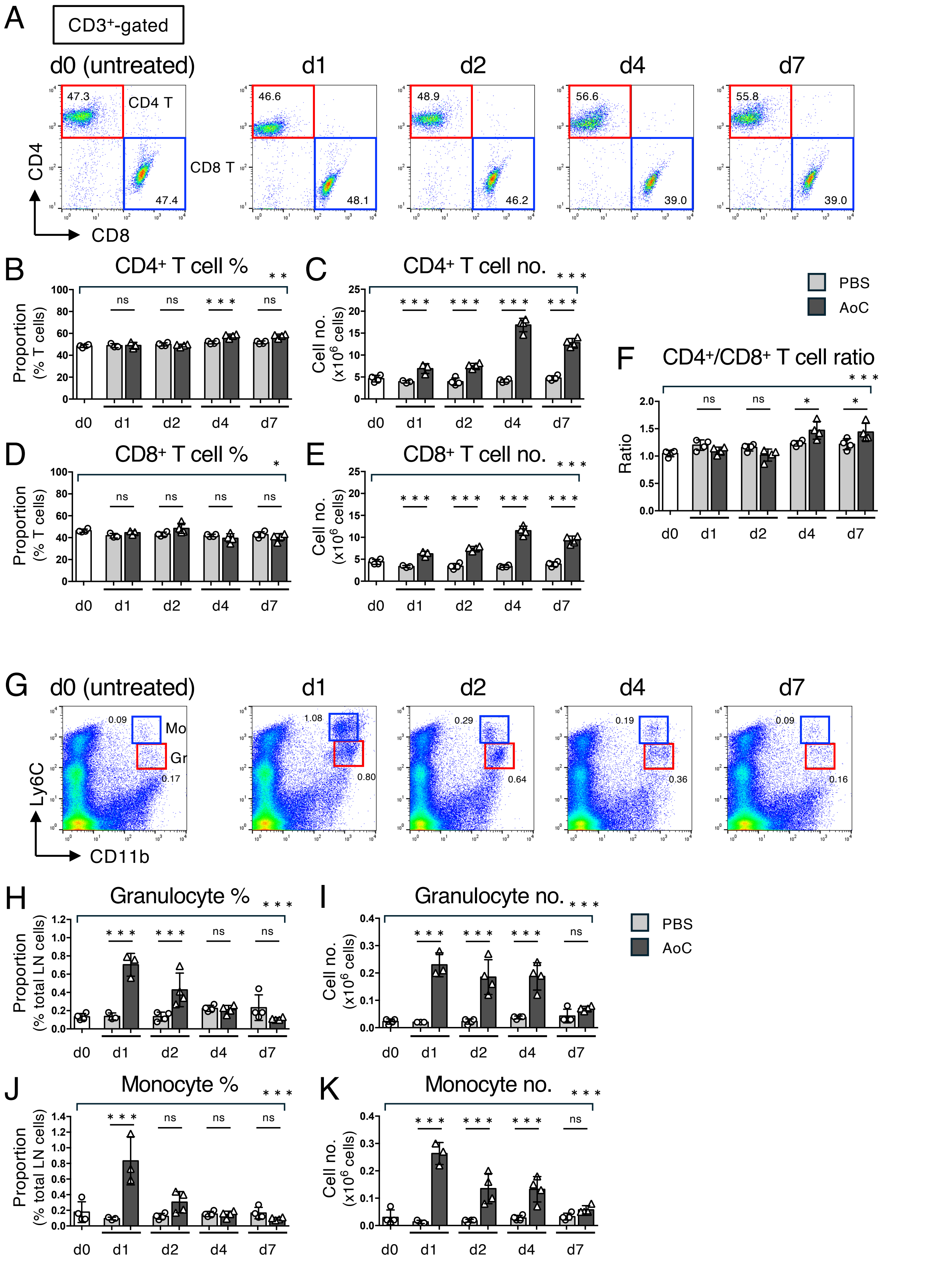

**Supplementary Figure 2.** AoC induces a change in T cell subsets and mobilization of myeloid cells in skin-draining LNs.

**(A)** Flow cytometric analysis for the composition of $CD4^+$ and $CD8^+$ T cells in LNs. Cells isolated from LNs at the indicated time points after AoC or PBS injection were stained for CD3, CD4, and CD8. $CD4^+$ and $CD8^+$ T cells were determined as $CD3^+CD4^+CD8^-$ (red) and $CD3^+CD4^-CD8^+$ (blue) gates. **(B-F)** Percentage changes **(B, D)** and numbers **(C, E)** of $CD4^+$ and $CD8^+$ T cells, and the ratio **(F)**. **(G)** Flow cytometric analysis for granulocytes and monocytes in LNs. Cells isolated from LNs at the indicated time points after AoC or PBS injection were stained for CD11b and Ly6C. Granulocytes (Gr) and monocytes (Mo) were determined as $CD11b^+Ly6C^{int}$ (red) and $CD11b^+Ly6C^{hi}$ (blue) gates. **(H-K)** Percentage changes **(H, J)** and numbers **(I, K)** of Gr and Mo. n= 4, Mean ± SD. Statistical analysis was performed using two-way ANOVA (upper, ⊓) with Sidak's post-test (lower, −). ns, not significant; $*p < 0.05$; $**p < 0.01$; $***p < 0.001$.

# Supplementary Figure 3

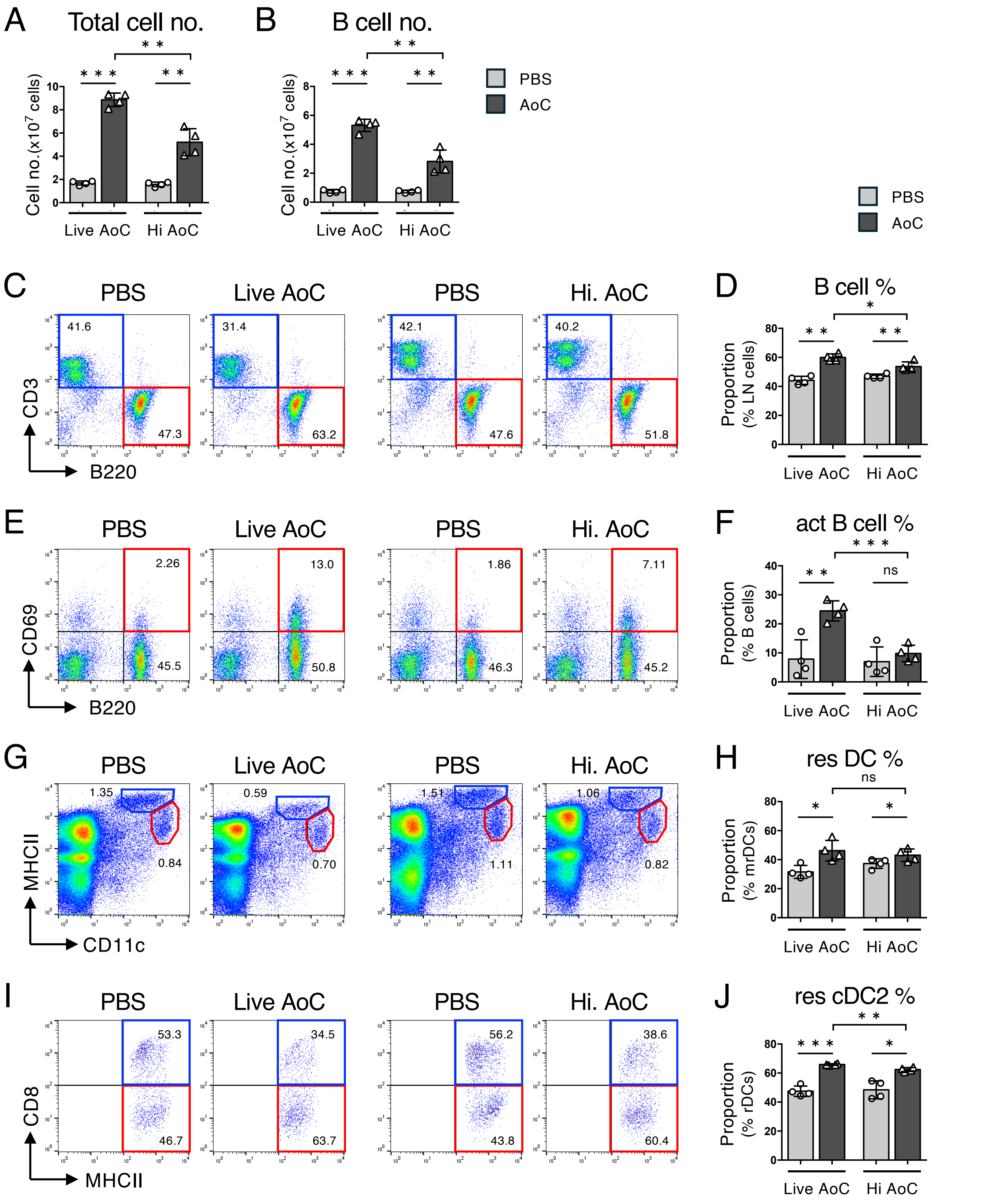

**Supplementary Figure 3.** Heat-inactivated AoC partially induces LN changes.

**(A, B)** Total cells **(A)** and B cells **(B)** in skin-draining LNs following the inoculation of live and heat-inactivated (Hi) AoC. **(C-J)** Flow cytometric analysis for the composition of B cells **(C, D)**, activated B cells **(E, F)**, res DCs **(G, H)**, and res cDC2 **(I, J)** in LNs following live and heat-inactivated AoC inoculation. n= 4, Mean ± SD. Statistical analysis was performed using an unpaired *t*-test (upper, ⊓) and a paired *t*-test (lower, −). ns, not significant; *$p < 0.05$; **$p < 0.01$; ***$p < 0.001$.

# Supplementary Figure 4

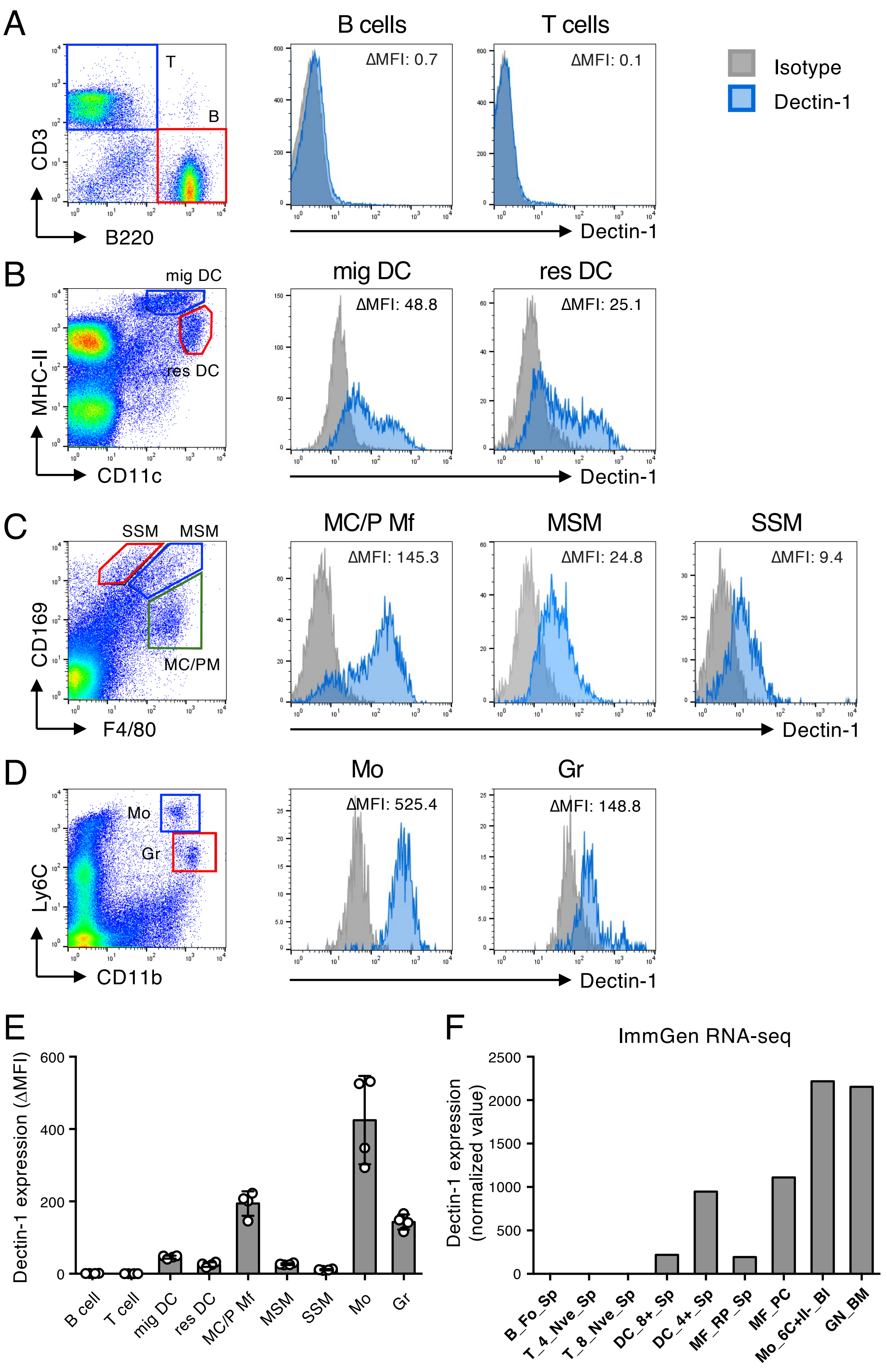

**Supplementary Figure 4.** Myeloid cells express Dectin-1 in skin-draining LNs.

(**A-D)** Flow cytometric analysis for detecting Dectin-1 expression on cell surface of lymphocyte subsets **(A)**, DC subsets **(B)**, macrophage subsets **(C)**, and blood leukocytes **(D)** in steady-state LNs. Dectin-1 and isotype control staining are shown in histograms. Delta mean fluorescence intensity (DMFI) is calculated by subtracting the MFI of isotype control from that of Dectin-1 staining. MC/P Mf, medullary cord/parenchymal macrophage; MSM, medullary sinus macrophage; SSM, subcapsular sinus macrophage. **(E)** Dectin-1 surface expression (DMFI) in LN. n= 4, Mean ± SD. **(F)** Transcriptional Dectin-1 expression in various immune cell subsets. Data were obtained from ImmGen RNA-seq database. Sp, spleen; PC, peritoneal cavity; Bl, blood; BM, bone marrow; Fo, follicular; Nve, naïve; RP, red pulp; MF, macrophage; GN, granulocyte; 4, CD4; 8, CD8; 6C, Ly6C; II, MHC-II.

# Supplementary Figure 5

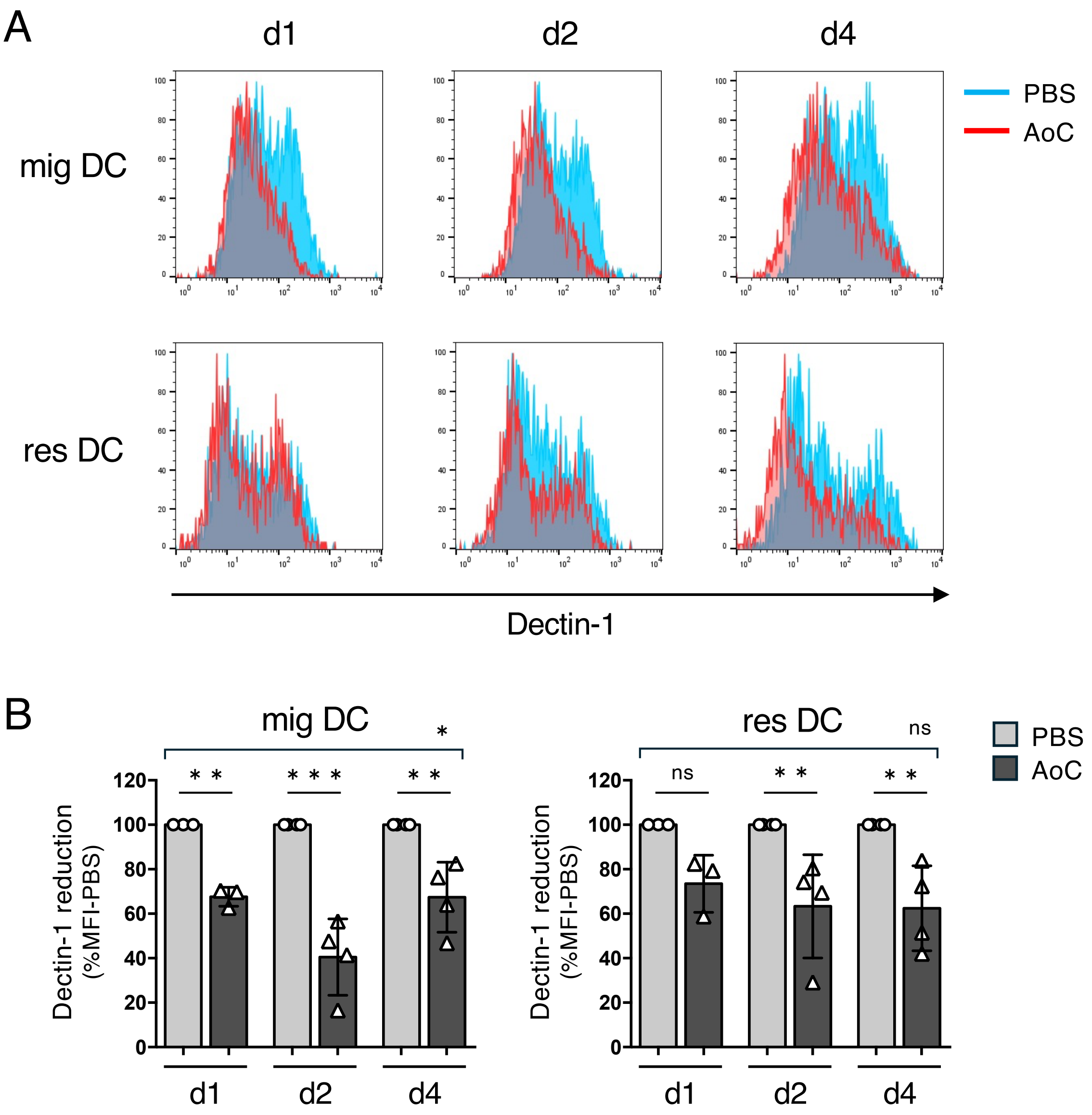

**Supplementary Figure 5.** AoC induces Dectin-1 down-regulation in DCs.

**(A)** Flow cytometric analysis for detecting Dectin-1 expression on DC subsets in skin-draining LN at indicated time points after AoC or PBS inoculation. Cell surface expressions of Dectin-1 in migratory (mig) DCs (upper) and resident (res) DCs (lower) are shown in histograms. **(B)** Dectin-1 reduction in mig DCs (left) and res DCs (right) is shown as the percentage of MFI in AoC inoculated side compared to the PBS side. n= 3 or 4, Mean ± SD. Statistical analysis was performed using two-way ANOVA (⊓) with Sidak's post-test (−). ns, not significant; *p < 0.05; **p < 0.01 ; ***p < 0.001.

# Supplementary Figure 6

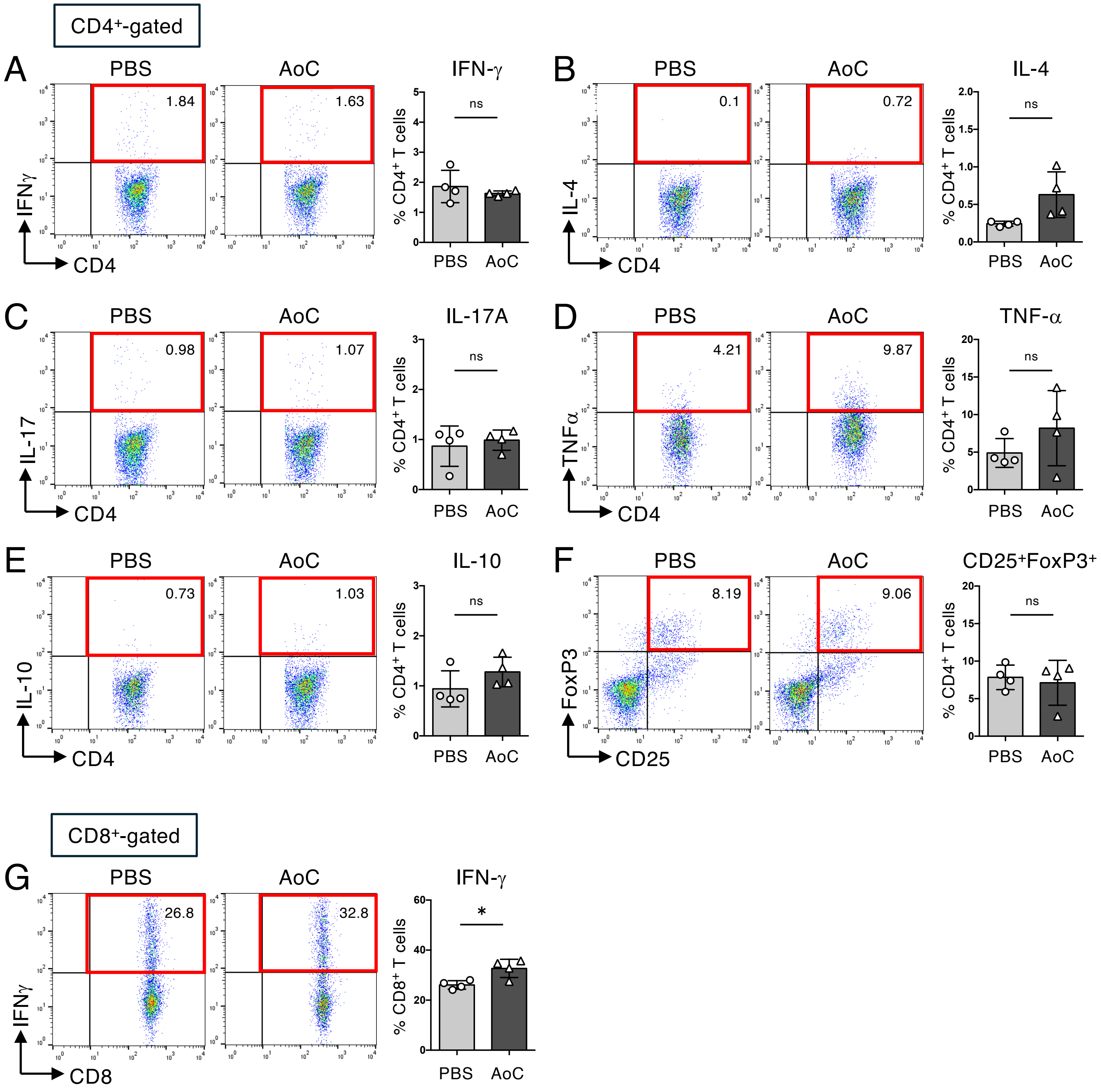

**Supplementary Figure 6.** AoC has a minimal effect on T cell polarization.

**(A-F)** Flow cytometric analysis for detecting cytokine production and Foxp3 expression by $CD4^+$ T cells in skin-draining LNs 7 days after AoC inoculation. **(G)** Flow cytometric analysis of IFN-γ production by $CD8^+$ T cells in skin-draining LNs 7 days after AoC inoculation. n = 4, Mean ± SD. Statistical analysis was performed using a paired *t*-test. ns, not significant; *$p < 0.05$.

# Supplementary Figure 7

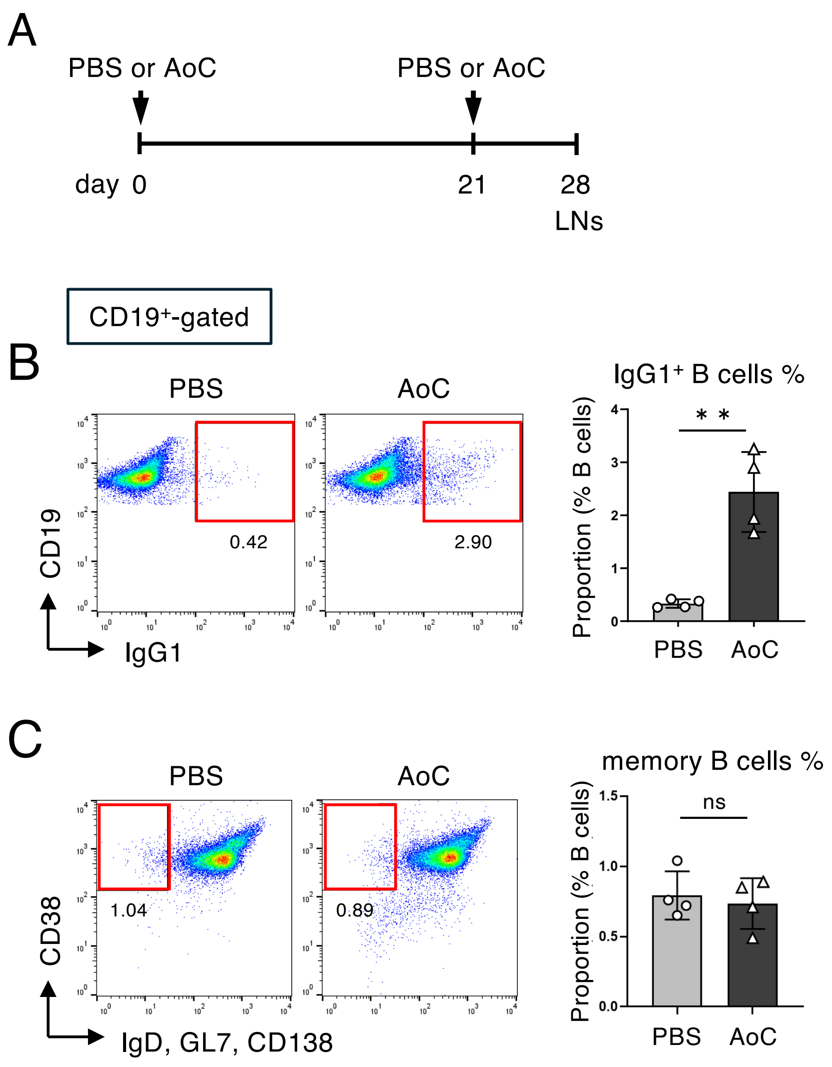

**Supplementary Figure 7.** AoC augments class-switched but not memory B-cell fraction.

**(A)** Scheme of AoC inoculation. **(B, C)** Flow cytometric analysis for detecting IgG1 class-switched B cells **(B)** and memory B cells **(C)**. Cells isolated from skin-draining LNs after AoC or PBS inoculation were stained for the indicated markers. Class-switched and memory B cells were determined as $CD19^{+}IgG1^{+}$ and $CD19^{+}IgD^{-}GL7^{-}CD138^{-}CD38^{+}$, respectively. $n = 4$, Mean ± SD. Statistical analysis was performed using a paired *t*-test. ns, not significant; $^{**}p < 0.01$ .
